# Autoantibodies targeting G protein-coupled receptors and clinical outcome after ischemic stroke (PROSCIS-B)

**DOI:** 10.1007/s00415-026-14103-6

**Published:** 2026-09-08

**Authors:** Anna-Sophia Miesenberger, Friederike A. Arlt, Leif Koschützke, Pimrapat Gebert, Harald Heidecke, Kai Schulze-Forster, Rusan Ali Catar, Guido Moll, Christoph Harms, Anna Kufner, Thomas Liman, Harald Prüss, Matthias Endres, Pia S. Sperber

**Affiliations:** 1 Center for Stroke Research Berlin (CSB), Charité-Universitätsmedizin Berlin, Berlin, Germany; 2 Department of Neurology and Experimental Neurology, Charité-Universitätsmedizin Berlin, Berlin, Germany; 3 German Center for Neurodegenerative Diseases (DZNE), Berlin, Germany; 4 Institute of Biometry and Clinical Epidemiology, Charité-Universitätsmedizin Berlin, Berlin, Germany; 5 CellTrend GmBH, Luckenwalde, Germany; 6 Department of Nephrology and Internal Intensive Care Medicine, Charité-Universitätsmedizin Berlin, Berlin, Germany; 7 BIH Center for Regenerative Therapies (BCRT), Berlin, Germany; 8 Julius Wolff Institute for Musculoskeletal Research (JWI), Berlin, Germany; 9 German Centre for Cardiovascular Research (DZHK), Berlin, Germany; 10 German Center for Mental Health (DZPG), Berlin, Germany; 11 Experimental and Clinical Research Center, A Cooperation Between Max Delbrück Center for Molecular Medicine in the Helmholtz Association, Charité-Universitätsmedizin Berlin, Berlin, Germany

**Keywords:** Stroke, Ischemia, Autoantibodies, G protein-coupled receptors, Epidemiology

## Abstract

**Background:**

G protein-coupled receptor (GPCR) directed regulatory autoantibodies (RABs) have previously been linked to poor functional outcome after ischemic stroke. We investigated the impact of anti-α1/α2/β1/β2 receptor, endothelin B receptor, angiotensin II receptor type 2, and CXCR3 receptor directed RABs on functional outcome and recurrent events after ischemic stroke.

**Methods:**

Data were derived from the Prospective Cohort with Incident Stroke Berlin (PROSCIS-B; NCT01363856). RABs were measured by ELISA (CellTrend) from sera collected within seven days of first-ever stroke. High RAB levels (quartile [Q]4 vs. Q1-3) and low levels (Q1 vs. Q2-4) were compared to reference groups. Quartile subgroups were characterized at baseline. Functional outcome, measured by the Modified Rankin Scale (mRS) at one year, was evaluated with ordinal logistic regression. Cox proportional hazard models were used to assess risk for a combined endpoint (stroke, myocardial infarction, death) over three years.

**Results:**

562 patients were included (mean age 67 (SD= 13); 38% female). All RABs showed more large artery atherosclerosis (LAA) stroke in Q1, while cardioembolic strokes (CE) were more frequent in Q4. Worse functional outcome at 12 months was associated with high levels (Q4) of alpha2-antibodies (abs) (OR=1.49; 95%CI=1.02–2.18) and ETB-abs (OR=1.49; 95%CI=1.02–2.18) vs. Q1-Q3. Recurrent cardiovascular risk over three years was higher with low levels (Q1) of AT2-abs (HR=1.86; 95%CI=1.22–2.86), alpha2-abs (HR=1.77; 95%CI=1.15–2.72), and beta1-abs (HR=1.58; 95%CI=1.03–2.44) vs. Q2-Q4.

**Conclusion:**

Differential associations of low vs. high levels of several RABs with stroke etiology, functional outcome, and recurrent vascular risk suggest potential roles in stroke pathophysiology.

**Supplementary Information:**

The online version contains supplementary material available at https://doi.org/10.1007/s00415-026-14103-6.

## Introduction

G protein-coupled receptors (GPCRs) constitute one of the largest receptor families, counting over 800 members with various functions [1]. Regulatory autoantibodies (RABs) directed against GPCRs can be detected in any individual, with unclear significance for both physiology and pathophysiology [2, 3]. RABs can impact the signaling of targeted receptors and function either as agonists or antagonists of their target GPCRs, depending on the respective binding site [2–4]. GPCRs are expressed in many tissues, relevant sites in the context of cardiovascular disease being endothelial cells and cardiomyocytes amongst others [4]. Thus, RABs are reported to influence vasoregulation by inducing pro-hypertensive and -atherogenic states, as demonstrated in experimental models and patient cohort studies [5–9]. Therefore, GPCRs and consequently RABs may influence several signaling pathways, including those involved in immune cell function, tissue invasion and local inflammatory milieu [10–12]. Consequently, the concept of the “antibodiome” was introduced, suggesting that an imbalance in either direction, i.e. elevated or reduced levels of RABs, may potentially influence the pathobiology of vascular disease [13, 14].

Differential levels of RABs (both low and high) have been identified in a range of rheumatological and cardiovascular conditions [2, 3]. Antibodies against adrenergic and angiotensin receptors, for example, have been associated with hypertension, arrhythmias, and cardiomyopathy [6, 15–21], while antibodies targeting chemokine or endothelin receptors have been linked to autoimmune and post-infectious diseases, such as systemic sclerosis, Sjögren syndrome, and Myalgic Encephalitis/Chronic Fatigue Syndrome (ME/CFS) [22–26]. In addition these findings, more recent studies suggest that RAB levels may serve as prognostic markers for cardiovascular events and mortality [5, 27]. Collectively, these observations point to a more extensive role of RABs in vascular pathology and prompt the inquiry of their potential impact on stroke outcomes.

Previously, also within in the PROSCIS-B cohort, RABs against complement factor 3a receptor and vascular endothelial growth factor receptor 2 were associated with poor functional outcome in ischemic stroke [28]. Here, we investigated a different set of RABs, namely alpha 1 adrenergic receptor antibodies (α1-abs), alpha 2 adrenergic receptor antibodies (α2-abs), beta 1 adrenergic receptor antibodies (β1-abs), beta 2 adrenergic receptor antibodies (β2-abs), angiotensin II receptor type 2 antibodies (AT2-abs), endothelin receptor type B antibodies (ETB-abs) and chemokine receptor 3 (CXCR3-abs). We investigated functional outcome at one year and risk of recurrent cardiovascular events over three years in patients with ischemic stroke. Moreover, we extended this analysis to two more outcomes of interest: cognitive outcome over three years and cerebral small vessel disease (CSVD) burden at baseline. By that, we aim to explore the broader implications of RABs in cerebrovascular disease.

## Methods

### Study design and patient population

The PROSCIS-B cohort (Prospective Cohort of Incident Stroke – Berlin) is an observational and hospital-based prospective clinical trial of first-ever stroke patients in Berlin, Germany. Recruitment took place at the three campuses of Charité – Universitätsmedizin Berlin hospitals. The study design has been described in detail previously [29]. Briefly, patients with incident stroke (i.e. ischemic stroke or brain hemorrhage or sinus vein thrombosis), as defined by the World Health Organization criteria were considered for study inclusion [30]. Male and female patients aged 18 or above were included and underwent clinical characterization within 7 days after admission. We excluded patients with prior diagnosis of brain tumor, brain metastasis, previous stroke, or participation in interventional studies. We further restricted the presented analysis to mild to moderate ischemic stroke patients, defined by National Institute of Health Stroke Scale (NIHSS) scores <16, to increase homogeneity of the study population as only few individuals with a severe stroke were included. Imaging was not part of the PROSCIS-B protocol and data, therefore, only obtained later.

### Antibody measurements

Within seven days of symptom onset, blood samples were drawn. Serum was separated by centrifugation at 1500 g for 15 min and aliquoted into 0.5 mL tubes after being left at room temperature for a maximum of 30–60 min. Samples were then stored at −80 °C and sent to collaboration partners at CellTrend laboratory (CellTrend GmbH, Luckenwalde, Germany) for antibody level determination. At CellTrend laboratory a sandwich enzyme-linked immunosorbent assay (ELISA)-Kit by CellTrend was used, allowing the measurement of antibodies of immunoglobulin G isotype (IgG) targeting α1, α2, β1, β2, AT2, ETB, and CXCR3 receptors. This ELISA identifies antibodies against epitopes and structures within the GPCR using whole-cell lysate from transfected Chinese hamster ovary cells overexpressing the respective human GPCR [3]. A standard curve is derived from a reference measurement with known concentration on each plate and used to define units (U/ml) of autoantibody concentrations from respective optical density measurements (curve over five standards; ranges are published in the respective product information for each kit on CellTrend website (https://www.celltrend.de/en/elisa/gpcr-antibodies/)).

### Exposure definition

In our analyses, each RAB was evaluated separately. Previous studies have suggested that both elevated as well as reduced levels of GPCR-directed autoantibodies may be associated with pathological processes (“antibodiome”) [13, 14], indicating that potential effects may not follow a linear relationship. To account for this possibility and to avoid imposing assumptions of linearity, we employed a quartile-based approach that has been commonly used in prior studies of GPCR autoantibodies, including previous analyses from the PROSCIS cohort. [28] Accordingly, low antibody levels, defined as the first quartile (Q1), were compared with patients in quartiles 2–4 (Q2–Q4), whereas high antibody levels, defined as the fourth quartile (Q4), were compared with patients in quartiles 1–3 (Q1–Q3). Thus, two separate analyses were performed for each antibody–outcome combination, allowing an assessment of potential associations in both directions.

### Outcome definition

In this exploration we considered four distinct outcomes at different timepoints to obtain a comprehensive view of the probable effects of these antibodies. First, we analyzed the functional outcome after one year using the modified Rankin Scale (mRS). The mRS is the most common instrument to assess functional outcome after stroke using 7 ordinal steps, from 0 meaning no disability to 6 representing death. It is validated for German language and telephone interviews [31, 32].

Second, we compared the time until the occurrence of a combined endpoint of stroke, myocardial infarction (MI) and all-cause death up to three years after the index event. The endpoints were obtained by postal or telephone contact (self-reported) on top of screening hospitals and treating physician records. Additionally, vital status was obtained from Berlin’s local registration office. All obtained endpoints were rated by two independent senior vascular neurologists for validation and only confirmed endpoints entered the analyses.

Third, we compared cognitive outcome over three years (contact after 1, 2 and 3 years for each patient) using the Telephone Interview for Cognitive Status modified (TICS-m) [33]. The TICS-m is a screening test for cognitive impairment with a maximum of 50 points (best performance), assessed by 20 questions [34].

Lastly, we compared cerebral small vessel disease burden (CSVD) assessed by Wahlund Score at baseline of exposure group (Q1 or Q4) with reference quartiles [35]. The Wahlund Score evaluates age-related white matter hyperintensities on cerebral computer tomography and magnetic resonance imaging scans. In this Score 5 predefined areas in both hemispheres are scored from 0 to 3 points, resulting in a total score of 0 to 30 [35].

### Statistical methods

Patients’ characteristics were summarized by means and medians with respective measures of variability (standard deviation [SD] and interquartile range boundaries [IQR] according to scale of measurement). To evaluate potential imbalances in baseline characteristics across subgroups, we calculated standardized mean differences (SMDs) for each variable. An SMD threshold of 0.1 was used to indicate potentially meaningful imbalance. SMD values were visualized using dot plots with variables ordered by effect size. To explore the relationship between antibody levels of different antibodies, pairwise correlations among autoantibody levels (U/mL) were assessed using Pearson’s correlation coefficients. To account for potential skewed distributions, log transformation was performed prior to correlation calculations. The resulting correlation matrix was visualized utilizing color-coded heatmaps.

For our main analysis, we performed separate confounder identification procedures using directed acyclic graphs implemented in a web application and software DAGitty [36, 37]. In this process confounders were defined as those variables with a potential effect on the exposure (i.e. the respective RAB) and the outcome of interest. Ultimately, we calculated three models for each outcome: one unadjusted, a second adjusted for age and sex only, and a third fully adjusted model in which we included all by respective DAG identified confounders (age, sex, and systemic inflammation as indicated by high sensitivity C-reactive protein [hsCRP]). The selection process of confounders for each model derived from DAGs can be found in Supplementary Fig. 1.

We plotted mRS with Grotta bars, stratified by exposure group. Functional outcome defined by mRS was analyzed using proportional odds regression models to estimate odds ratios (OR) for the exposure group compared to comparator quartiles [38]. Because of the limited numbers in the high mRS categories (5 and 6 points), we pooled these two categories to ensure sufficient observations in each category.

Event-free (i.e. stroke recurrence, myocardial infarction, or all-cause death) survival time was calculated in person-years (py). Survival time was censored at the occurrence of an event, end of study or time or last contact, depending on which occurred sooner. Incidence rates were calculated to estimate the events per 100 person years. Cumulative hazards were visualized using Kaplan–Meier estimates transformed as –log(S(t)) to reflect increasing event risk over time. We then calculated Cox proportional hazard regression models to estimate hazard ratio (HR) over 3 years after first stroke [39].

We computed a linear mixed model (lmm) with cognitive outcome as measured by TICS-m as the dependent variable. The lmm included patient-ID as a random effect to account for repeated measurements over time (autocorrelated observations). Time and the RABs (i.e., Q1 or Q4 vs. Q2-4 or Q1-3, respectively) were included as fixed effects, as well as confounders.

Lastly, to estimate the potential impact of respective autoantibody levels on CSVD burden, we calculated a linear regression including the Wahlund Score. Additionally, to consider a more pragmatic application of the Wahlund Score, we also compared high to low CSVD burden by calculating a logistic regression using a dichotomized outcome (Scores <10 or ≥10), in line with previous analysis in PROSCIS-B [40]. An alpha level of 0.05 was used to determine statistical significance for ease of interpretation for all analyses.

Additionally, we conducted a post-hoc analysis to assess whether the results we obtained were influenced by stroke etiology as defined by TOAST to not overlook a potential association between stroke etiology and functional outcome as well as recurrent risk [41]. For that, we divided the overall cohort into subgroups of interest, namely large artery atherosclerosis (LAA) and cardioembolic (CE) strokes. First, we evaluated the impact of LAA and CE strokes on functional outcome and recurrent risk. We then assessed the impact of high (Q4) and low (Q1) antibodies against the remainder within each etiological subgroup. Both analyses were done using the same models (proportional odds logistic regression and Cox proportional hazard model), and confounder selection (fully adjusted model 3 – age, sex, systemic inflammation) that were used for the overall cohort as described in detail above. Moreover, we conducted post-hoc causal mediation analyses (using the mediate function from the R “mediation” package) to detangle the direct effect of antibody levels from the indirect effect mediated via specific stroke etiology. We fitted the mediation in line with the primary analyses, however, for the combined endpoint mediation analysis, we fitted parametric survival regression models using a Weibull distribution (“survreg” in R) as the Cox proportional hazard model is not suitable for mediation analyses in this package.

Additionally, we also explored medication intake in all RAB subgroups over 3 years in a post-hoc analysis to assess distribution among subgroups and, therefore, potential influence of medication intake on outcome or antibody level utilizing descriptive statistics.

We furthermore conducted a post-hoc dose–response analysis for the functional outcome and the recurrent risk, where we used Q1 as a reference and compared each of the other quartiles individually to Q1 for the fully stratified Model 3.

### Computational tools and data availability

All statistical analyses and data visualizations were conducted using R (R version 4.4.0 (2024–04–24)) within the RStudio environment. Visualization of the causal concept for confounder identification was performed with the web application of DaGitty (https://dagitty.net/dags.html). All data and analysis scripts are available upon reasonable request from the principal investigator of PROSCIS-B (Prof. Matthias Endres, matthias.endres@charite.de).

## Ethics approval

Informed written consent was given by either patients or their legal representatives. PROSCIS-B was approved by the Ethics Committee of the Charité (EA1/218/09) and was conducted in line with the principles of the Declaration of Helsinki.

## Results

### Study population

**Fig. 1:**
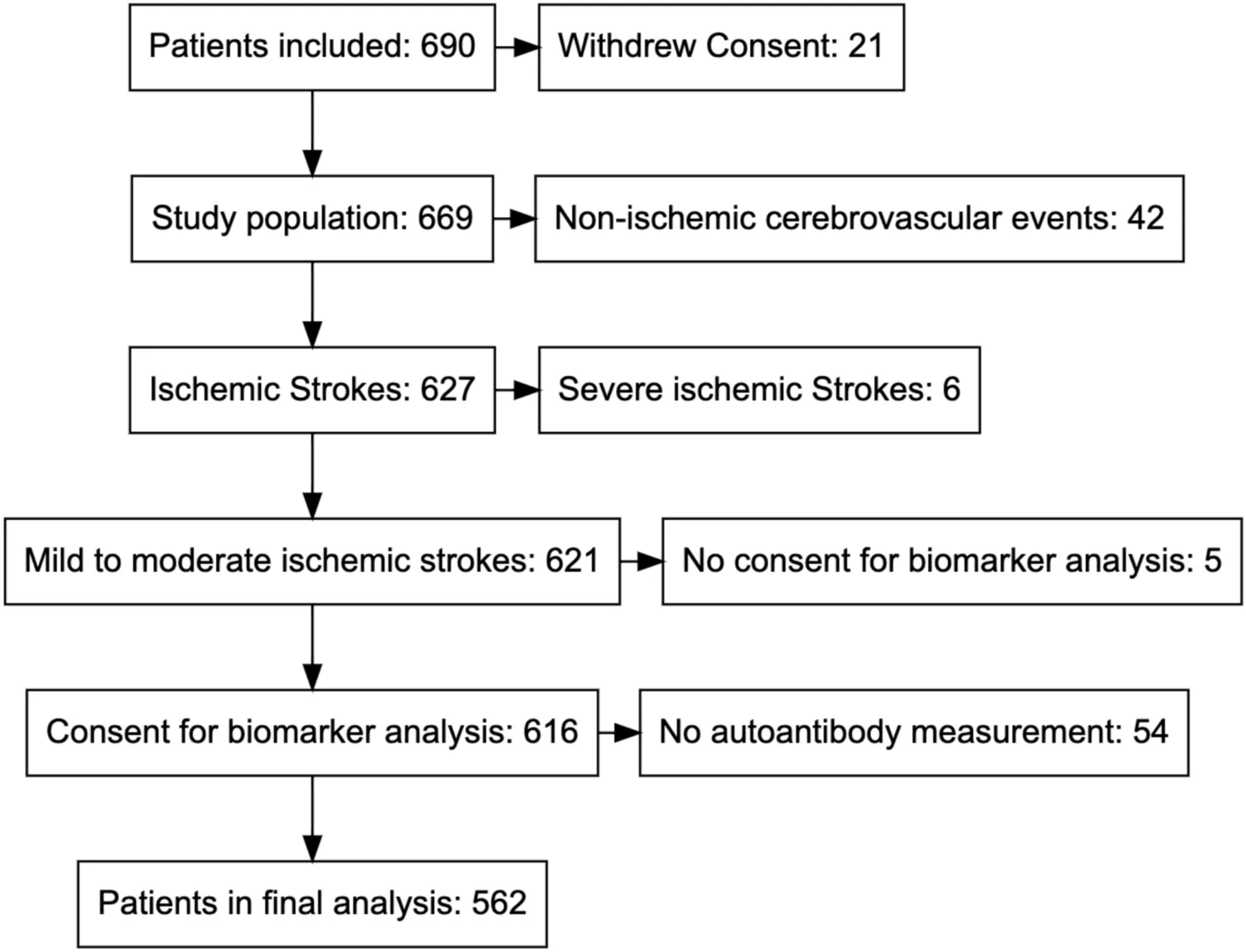
Study inclusion Flowchart. Flowchart illustrating study inclusion and exclusion of PROSCIS-B. Exclusion under “no consent” occurred when no written informed consent was available. NIHSS stands for National Institute of Health Stroke Scale. PROSCIS-B stand for Prospective Cohort with Incident Stroke Berlin

Overall, 621 ischemic stroke patients with an NIHSS <16 were recruited between 2010 and 2013. A total of 562 patients (90%) with complete antibody measurements were considered for our analysis. Serum samples were collected between day 3 and day 7 after the incident stroke. We excluded individuals because consent for biomarker analysis was unavailable, samples were damaged, or other technical issues occurred. Consequently, the number of patients included in the present analysis differs from that reported by Liman et al., reflecting both the different outcomes investigated and variations in the number of blood samples available as the antibody panel presented here was measured at a later time point [28]. Details on inclusion can be found in the flowchart in Fig. 1.

### Baseline characteristics and cross-sectional analyses

**Fig. 2:**
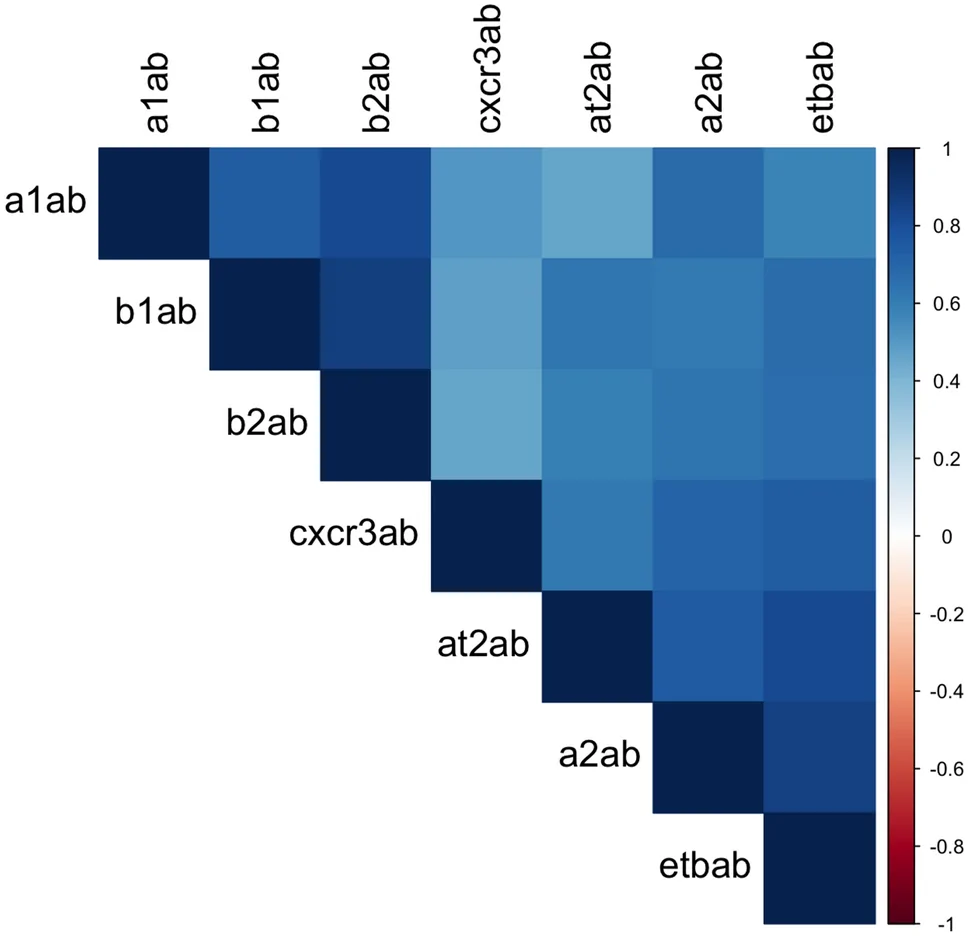
Antibody correlation plot. Correlation plot of RABs. *a1ab* alpha-1 adrenergic receptor antibodies, *a2ab* alpha-2 adrenergic receptor antibodies, *b1ab* beta-1 adrenergic receptor antibodies, *b2ab* beta-2 adrenergic receptor antibodies, *at2ab* angiotensin II receptor type 2 antibodies, *etbab* endothelin receptor type B antibodies, *cxcr3ab* chemokine receptor CXCR3 antibodies

Table 1 shows the clinical characteristics of our study cohort. Patients had a mean age of 67 (SD= 13) and 215 were female (38%). 153 patients (27%) were active smokers at study inclusion. Patients had a median NIHSS at admission of 2 (IQR=1–4). Clinical characteristics stratified upon all antibody quartile subgroups are shown in Supplementary Table 1. We observed a positive correlation among antibody levels, showing that elevated levels of one antibody were often associated with higher levels of others, as shown by the correlation plot in Fig. 2. In contrast, no negative correlations (higher levels of one RAB typically associated with lower levels of another RAB) were observed. There were higher values of inflammatory markers (IL-6 and hs-CRP) for patients in Q4 and lower values in Q1 for all RABs (the only exception being α1-abs: mean hs-CRP values in Q4 were not higher compared to Q1). Standardized mean differences (SMD) for the different subgroups were calculated and visualized (see Supplementary Figs. 2 and 3). Distribution of stroke etiology as classified by Trial of Org 10,172 in Acute Stroke Treatment (TOAST) differed between low (Q1) and high (Q4) antibody levels for all antibodies [41], showing higher numbers of large artery atherosclerosis (LAA) in patients with RAB levels in Q1 while more cardioembolic strokes (CE) occurred in patients with RAB levels in Q4. The distribution of stroke etiology for all RAB level subgroups is shown in Table 2. There were more patients treated with thrombolysis in Q1 as compared to Q4 for all RABs. No notable other differences or patterns could be detected between quartile subgroups at baseline. Supplementary Table 2 shows the distribution of medication use across all quartile subgroups. We accounted differences in medication intake for beta-blockers and lipid lowering therapeutics for all RABs studied as well as ACE-inhibitors/AT-blockers (with the exception of β1-abs and β2-abs) which were more frequently administered in Q1 as compared to Q4. These differences were already present at study inclusion and persisted over the duration of the study. We also observed differences in antiplatelet therapy, which was more frequent in Q1 for all RABs when compared to Q4. In line with that, when looking at medication intake in subgroups classified by TOAST we accounted for a higher number of antiplatelet therapy in LAA strokes while a higher number of patients with a CE stroke were anticoagulated in follow-up visits (data not shown).

**Table 1:** Baseline characteristics

	Overall (*N*=562)
Age mean (SD)	67.0 (12.8)
Age group *n* (%)	
<55	96 (17)
55–64	124 (22)
65–74	179 (31)
>74	163 (29)
Sex *n* (%)	
Female	215 (38)
Regular alcohol consumption *n* (%)	200 (36)
Active smoking *n* (%)	153 (27)
Body mass index mean (SD)	27.5 (5.01)
Systolic blood pressure (mmHg) mean (SD)	139 (22.3)
Diastolic blood pressure (mmHg) mean (SD)	76.8 (14.3)
Total cholesterol mean (SD)	199 (48.3)
Low density lipoprotein mean (SD)	123 (40.7)
NIHSS at admission	
Median [Q1, Q3]	2.00 [1.00, 4.00]
NIHSS ranges *n* (%)	
0–4	428 (76)
5–15	134 (24)
Stroke etiology (TOAST) *n* (%)	
Large artery artherosclerosis (LAA)	146 (26)
Cardioembolic (CE)	132 (24)
Small artery occlusion (SAO)	91 (16)
Other cause	16 (3)
Undefined	177 (32)
Preexisting cardiovascular conditions *n* (%)	
Diabetes	120 (21)
Hypertension	368 (66)
Atrial fibrillation	119 (21)
Peripheral arterial disease	39 (7)
Myocardial infarction	18 (3)
Angina pectoris	87 (16)
Treatment *n* (%)	
Thrombolysis	113 (20)
Thrombectomy	8 (1)
high sensitivity CRP	
Mean (SD)	13.0 (21.6)
Interleukin 6	
Mean (SD)	10.8 (45.5)
Interleukin 6 negative *n* (%)	223 (40)
Interleukin 6 positive *n* (%)	323 (58)
RABs median [Q1, Q3]	
Alpha1-abs	9.39 [7.00, 12.7]
Alpha2-abs	11.4 [7.67, 20.1]
Beta1-abs	13.0 [8.98, 19.3]
Beta2-abs	10.8 [7.07, 17.7]
AT2-abs	6.60 [4.20, 11.7]
ETB-abs	15.0 [9.86, 27.2]
CXCR3-abs	29.2 [16.8, 48.9]
Wahlund score	
Mean (SD)	6.57 (5.42)
Wahlund–severity *n* (%)	
Mild (0–9)	289 (51)
Severe (≥ 10)	93 (17)
Missing	180 (32)

Baseline characteristics of the overall study cohort. Rounding might result in percentages not summing up to 100%

*NIHSS* National institute of health stroke scale, *TOAST* Trial of ORG 10172 in acute stroke treatment, *CRP* C-reactive protein, *RABS* regulatory antibodies; alpha1-abs, *alpha-1* adrenergic receptor antibodies, *alpha2-abs* alpha-2 adrenergic receptor antibodies, *beta1-abs* beta-1 adrenergic receptor antibodies, *beta2-abs* beta-2 adrenergic receptor antibodies, *AT2-abs* angiotensin II receptor type 2 antibodies, *ETB-abs* endothelin receptor type B antibodies, *CXCR3-abs* chemokine receptor CXCR3 antibodies

**Table 2:** Distribution of TOAST stroke etiology across antibody quartiles (Q1 vs Q4)

TOAST	LAA *n (%)*		CE *n (%)*		SAO *n (%)*		Other *n (%)*		Undefined *n (%)*	
Sub-group	Q1	Q4	Q1	Q4	Q1	Q4	Q1	Q4	Q1	Q4
Alpha1	50 (36)	27 (19)	30 (21)	41 (29)	19 (14)	21 (15)	2 (1)	7 (5)	40 (28)	45 (32)
Alpha2	45 (32)	27 (19)	36 (26)	41 (29)	19 (14)	22 (16)	5 (4)	9 (6)	36 (26)	42 (30)
Beta1	42 (30)	36 (26)	38 (27)	42 (30)	23 (16)	18 (13)	1 (1)	8 (6)	37 (26)	37 (26)
Beta2	51 (36)	32 (23)	29 (21)	39 (28)	22 (16)	18 (13)	3 (2)	7 (5)	36 (26)	45 (32)
AT2	42 (30)	33 (23)	32 (23)	42 (30)	19 (14)	24 (17)	3 (2)	7 (5)	45 (32)	35 (25)
ETB	42 (30)	24 (17)	33 (23)	41 (29)	24 (17)	26 (18)	3 (2)	9 (6)	39 (28)	41 (29)
CXCR3	49 (35)	28 (20)	36 (26)	37 (26)	13 (9)	25 (18)	2 (1)	7 (5)	41 (29)	44 (31)

*LAA* large-artery atherosclerosis, *CE* cardioembolic, *SAO* small-artery occlusion, *other* stroke of other determined cause, *undefined* stroke of undetermined cause, *alpha1, alpha-1* adrenergic receptor antibodies, *alpha2* alpha-2 adrenergic receptor antibodies, *beta1* beta-1 adrenergic receptor antibodies, *beta2* beta-2 adrenergic receptor antibodies, *AT2* angiotensin II receptor type 2 antibodies, *ETB* endothelin receptor type B antibodies, *CXCR3* chemokine receptor CXCR3 antibodies, *Q* Quartile

### Functional outcome at one year

**Fig. 3:**
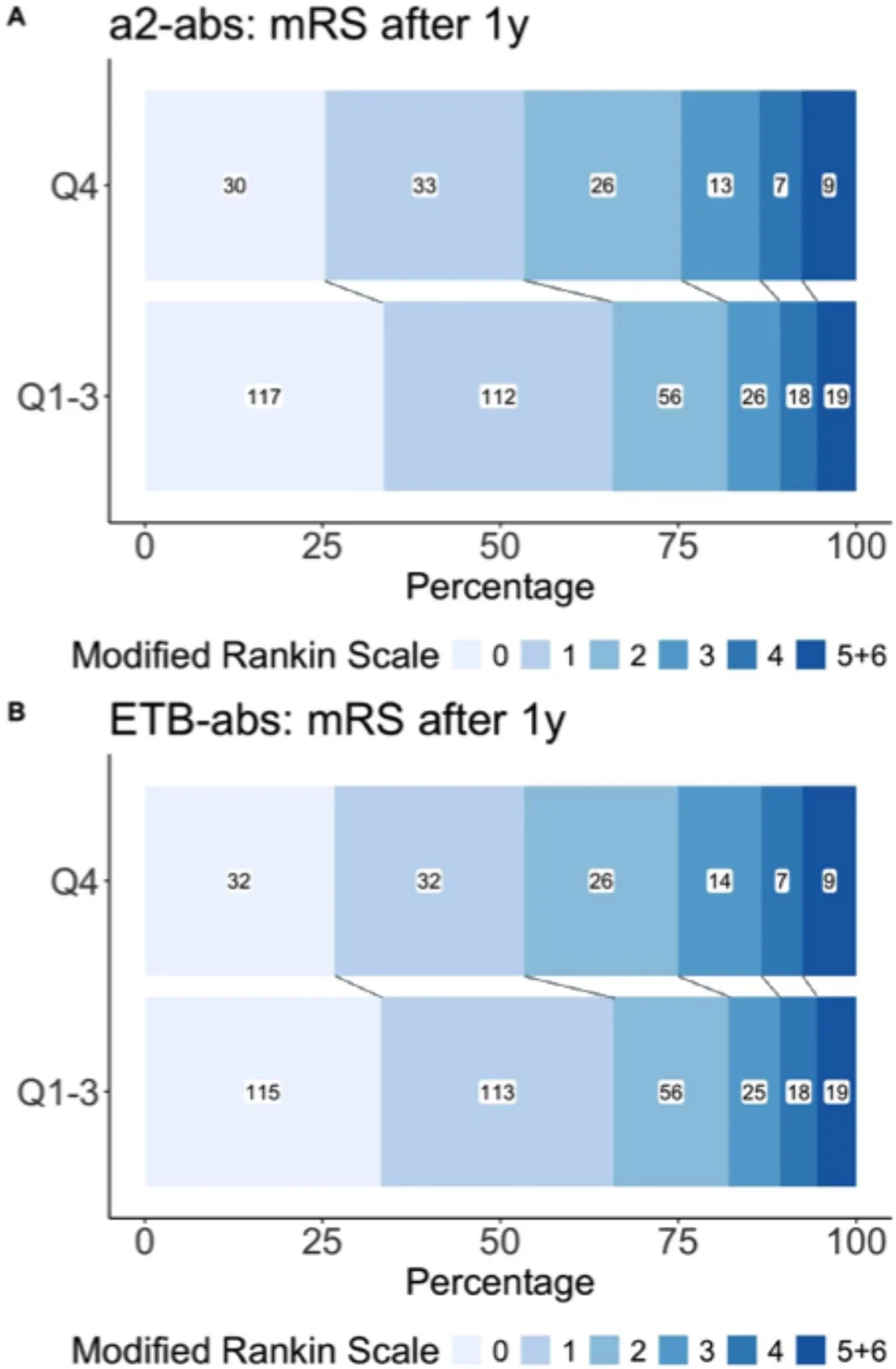
Modified Rankin Scale one year after stroke. Grotta bar plots visualizing difference functional outcome (mRS, modified Rankin Scale) after one year. mRS categories 5 + 6 were collapsed into one category. **A** Visualization of alpha-2 adrenergic receptor antibodies Quartile 4 vs. Quartile 1–3. **B** Visualization of endothelin receptor type B antibodies Quartile 4 vs. Quartile 1–3

The mRS at one year was available for 466 (83%) patients. The median mRS was 1 (IQR= 0–2) and did not differ between patients with high and low levels of respective RABs. Figure 3 shows mRS plotted stratified by RAB levels of Q4 vs Q1-3, which showed statistically significant differences in the odds models for ETB- and α2-abs. Visualizations of mRS for the remaining antibodies (ie. α1-abs, β1-abs, β2-abs, AT2-abs, CXCR3-abs) for Q4 vs. Q1-3 comparisons can be found in Supplementary Fig. 4 as well as visualization of mRS for all antibodies with levels in Q1 vs. Q2-4 of all RABs in Supplementary Fig. 5. Statistically significant differences were observed when comparing Q4 of ETB- (OR=1.49; 95%CI=1.02–2.18) and alpha2-antibodies (OR=1.49; 95%CI=1.02–2.18) to Q1-3 with proportional odds models. These antibodies also showed a dose–response relation in our post-hoc analysis (see Supplementary Table 3). A sensitivity analysis including thrombolysis as a confounder did not change the obtained results (data not shown). Mediation analysis showed no indirect effect through CE strokes. However, direct effects were observed, with Q4 antibody levels being associated with a lower probability of full recovery (mRS = 0; −8 percentage points for both α2 and ETB antibodies) and a higher probability of poor functional outcome (mRS = 5–6; +2.3 percentage points for both antibodies). Details can be viewed in Supplementary Fig. 6. Low levels of any antibody were not associated with functional outcome in any model. Full results can be viewed in Table 3. The proportional odds assumption was violated for α1, α2, β2, AT2 and ETB in Q1; β1, β2 and AT2 in Q4, meaning the direction of the association varied depending on the chosen modified Rankin Scale (mRS) cut-off. Therefore, an overall estimate renders non-meaningful results in this case. However, in an additional dichotomized model using a cut-off of mRS > 1 the associations remained largely unchanged (data not shown). We did not observe an impact of LAA and CE strokes on functional outcome (OR=0.94; 95%CI=0.63–1.40 and OR=1.11; 95%CI=0.77–1.61, respectively). Within the subgroups of LAA and CE strokes, we did not observe an association of antibody levels and outcome. Details can be found in Supplementary Table 4.

**Table 3:** Functional outcome after 1 year assessed by modified rankin scale

Antibody target	Model 1		Model 2		Model 3		
	OR	95%CI	OR	95%CI	OR	95%CI	*p*
Alpha 1							
Q1 vs Q2-4	0.89	(0.61–1.31)	0.82	(0.56–1.21)	0.82	(0.56–1.21)	0.32
Q4 vs Q1-3	0.80	(0.54–1.17)	0.82	(0.56-1.21)	0.85	(0.57–1.25)	0.40
Alpha 2							
Q1 vs Q2-4	0.79	(0.54–1.15)	0.76	(0.52–1.11)	0.83	(0.57–1.22)	0.35
Q4 vs Q1-3	1.54	(1.06–2.25)	1.59	(1.09–2.32)	1.49	(1.02–2.18)	0.03
Beta 1							
Q1 vs Q2-4	0.80	(0.55–1.17)	0.77	(0.52–1.11)	0.77	(0.53–1.13)	0.18
Q4 vs Q1-3	1.08	(0.73–1.59)	1.04	(0.70–1.53)	0.96	(0.65–1.42)	0.84
Beta 2							
Q1 vs Q2-4	1.02	(0.70–1.48)	0.95	(0.65–1.38)	0.92	(0.63–1.35)	0.68
Q4 vs Q1-3	0.99	(0.67–1.44)	1.01	(0.68–1.48)	0.93	(0.62–1.36)	0.70
AT2							
Q1 vs Q2-4	1.00	(0.69–1.45)	0.98	(0.67–1.42)	1.05	(0.72–1.53)	0.74
Q4 vs Q1-3	1.38	(0.94–2.01)	1.36	(0.93–1.99)	1.33	(0.91–1.94)	0.12
ETB							
Q1 vs Q2-4	0.86	(0.59–1.25)	0.81	(0.56–1.18)	0.84	(0.58–1.23)	0.38
Q4 vs Q1-3	1.51	(1.04–2.19)	1.53	(1.05–2.23)	1.49	(1.02–2.18)	0.04
CXCR3							
Q1 vs Q2-4	0.77	(0.53–1.12)	0.73	(0.50–1.06)	0.76	(0.52–1.11)	0.64
Q4 vs Q1-3	1.21	(0.83–1.77)	1.19	(0.81–1.73)	1.18	(0.80–1.72)	0.98

Functional outcome one year after first ischemic stroke assessed by mRS (modified Rankin Scale)

*OR* Odds Ratio from the proportional odds logistic regression, *95%-CI* 95% Confidence interval, *Model 1* unadjusted crude model, *Model 2* adjusted for age and sex, *Model 3* adjusted for age, sex and systemic inflammation (hsCRP), *alpha1-abs* alpha-1 adrenergic receptor antibodies, *alpha2-abs* alpha-2 adrenergic receptor antibodies, *beta1-abs* beta-1 adrenergic receptor antibodies, *beta2-abs* beta-2 adrenergic receptor antibodies, *AT2-abs* angiotensin II receptor type 2 antibodies, *ETB-abs* endothelin receptor type B antibodies, *CXCR3-abs* chemokine receptor CXCR3 antibodies, *Q* Quartile

### Recurrent cardiovascular risk over three years

**Fig. 4:**
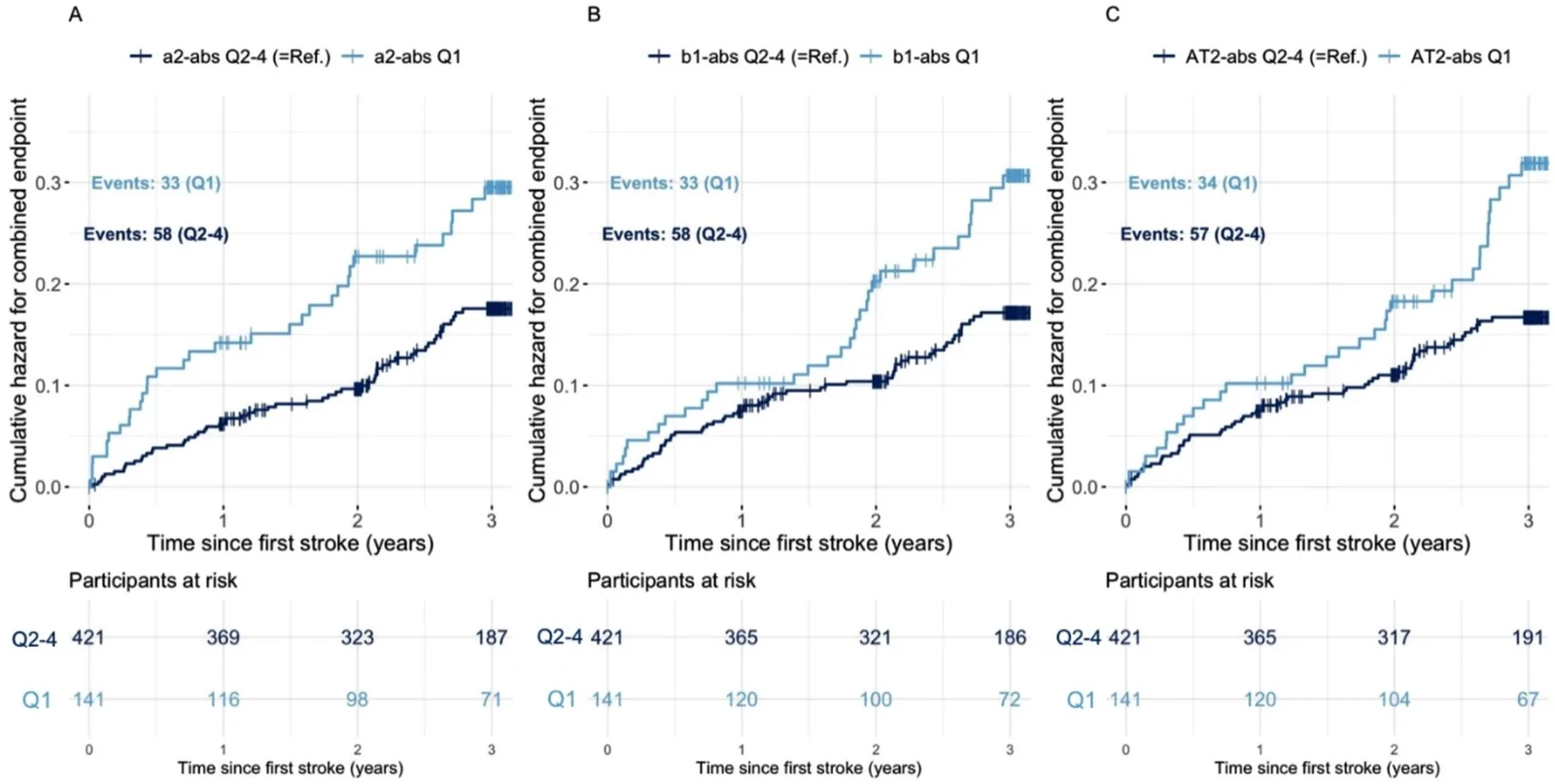
Cumulative Hazard over three years after ischemic stroke. Cumulative hazard functions over three years after incident ischemic stroke for a combined endpoint (recurrent stroke, myocardial infarction, all-cause death). Vertical lines on the graph represent an event. **A** Patients with alpha-2 adrenergic receptor antibodies in Quartile (Q)1 vs. Q2-4. **B** Patients with beta-1 adrenergic receptor antibodies in Q1 vs. Q2-4. **C** Patients with angiotensin II receptor type 2 antibodies in Q1 vs. Q2-4

Recurrent cardiovascular events or death were observed in 91 of 562 (16%) patients over a total follow up time of 1390 person years. Out of those events, 41 patients (7%) experienced a recurrent stroke, four patients (1%) a myocardial infarction, and 46 patients (8%) died within a follow-up period of three years. This resulted in an incidence rate of 6.5 events per 100 person years. We observed a higher incidence rate for all RABs in Q1 as compared to Q4; details can be found in Supplementary Tab. 5. The Kaplan–Meier curves undermined these observed differences in incidence rates (see Fig. 4 and Supplementary Fig. 7 for Q1 as well as Supplementary Fig. 8 for Q4). Fully adjusted Cox regression models (model 3) estimated an increased hazard for patients with antibody levels in Q1 of AT2-abs (HR=1.86; 95%CI=1.22–2.86), alpha2-abs (HR=1.77; 95%CI=1.15–2.72), and beta1-abs (HR=1.58; 95%CI=1.03–2.44) vs. reference groups (Q2–Q4) (Table 4). A sensitivity analysis including thrombolysis as a confounder did not change the obtained results (data not shown). Mediation analysis of a parametric survival regression model did not reveal mediation through LAA stroke of these results for all 3 antibodies. Visualization can be viewed in Supplementary Fig. 9. The impact of LAA (HR=1.26; 95%CI=0.80–2.00) and CE (HR=0.93; 95%CI=0.58–1.50) strokes on recurrent risk for the combined endpoint was not statistically significant. Within the group of patients with a CE stroke, we observed an association between low levels (Q1) of alpha2 (HR=2.34; 95%CI=1.05–5.24), beta2 (HR=2.31; 95%CI=1.02–5.23), and ETB (HR=2.30; 95%CI=1.04–5.09) antibodies with the combined endpoint. On the contrary, in the LAA subgroup an association was observed for high levels (Q4) of CXCR3 (HR=2.69; 95%CI=1.15–6.32). Details can be found in Supplementary Table 6. A post-hoc dose–response analysis can be viewed in Supplementary Table 7.

**Table 4:** Hazard ratios for a combined endpoint over 3 years

Antibody target	Model 1		Model 2		Model 3		
	HR	95%CI	HR	95%CI	HR	95%CI	*p*
Alpha 1							
Q1 vs Q2-4	1.35	(0.87–2.11)	1.17	(0.75–1.83)	1.15	(0.73–1.81)	0.54
Q4 vs Q1-3	0.68	(0.40–1.15)	0.76	(0.45–1.30)	0.77	(0.45–1.31)	0.34
Alpha 2							
Q1 vs Q2-4	1.76	(1.15–2.70)	1.69	(1.10–2.60)	1.77	(1.15–2.72)	0.01
Q4 vs Q1-3	0.95	(0.59–1.54)	1.05	(0.65–1.69)	1.04	(0.64–1.68)	0.89
Beta 1							
Q1 vs Q2-4	1.75	(1.14–2.68)	1.62	(1.05–2.48)	1.58	(1.03–2.44)	0.04
Q4 vs Q1-3	0.86	(0.53–1.41)	0.83	(0.51–1.36)	0.81	(0.50–1.33)	0.40
Beta 2							
Q1 vs Q2-4	1.62	(1.05–2.51)	1.49	(0.97–2.31)	1.46	(0.94–2.27)	0.09
Q4 vs Q1-3	0.80	(0.49–1.32)	0.87	(0.53–1.44)	0.85	(0.52–1.41)	0.53
AT2							
Q1 vs Q2-4	1.80	(1.18–2.75)	1.81	(1.18–2.77)	1.86	(1.22–2.86)	0.004
Q4 vs Q1-3	0.70	(0.42–1.18)	0.67	(0.40–1.13)	0.66	(0.39–1.11)	0.12
ETB							
Q1 vs Q2-4	1.52	(0.99–2.35)	1.42	(0.92–2.19)	1.46	(0.94–2.26)	0.09
Q4 vs Q1-3	0.81	(0.49–1.33)	0.83	(0.51–1.37)	0.83	(0.51–1.37)	0.48
CXCR3							
Q1 vs Q2-4	1.24	(0.79–1.94)	1.08	(0.69–1.70)	1.11	(0.71–1.75)	0.16
Q4 vs Q1-3	1.00	(0.62–1.60)	1.00	(0.62–1.61)	1.01	(0.63–1.62)	0.40

Hazard ratios of high and low antibody-levels for the respective antibody

*Model 1* unadjusted crude model, *Model 2* adjusted for age and sex, *Model 3* adjusted for age, sex and systemic inflammation (hsCRP), *HR* hazard ratio, *95% CI* 95% confidence interval, *Q1* measurements within the first quartile of antibody levels, *alpha1-abs* alpha-1 adrenergic receptor antibodies, *alpha2-abs* alpha-2 adrenergic receptor antibodies, *beta1-abs* beta-1 adrenergic receptor antibodies, *beta2-abs* beta-2 adrenergic receptor antibodies, *AT2-abs* angiotensin II receptor type 2 antibodies, *ETB-abs* endothelin receptor type B antibodies, *CXCR3-abs* chemokine receptor CXCR3 antibodies, *Q* Quartile

### Cognitive outcome over three years

Data from cognitive testing with TICS-m was missing from 190 (33%), 252 (45%) and 294 (52%) after one, two, and three years, respectively, in the overall study population. The mean value increased from 33 (SD 5) to 35 (SD 5) from the first assessment (after one year) to the last assessment (after three years), showing improvement of cognitive testing over time. Differences in TICS-m between patients with high vs. low levels of antibodies were very small or not statistically significant (e.g., α2-abs Q4 vs. Q1-3: beta = − 1.07, 95%CI= − 2.13–− 0.02; AT2-abs Q4 vs. Q1-3: beta = − 1.00, 95%CI= − 2.03–0.02). Detailed results from linear mixed models are shown in Supplementary Table 8.

### Cerebral small vessel disease burden (CSVD)

An assessment of CSVD at baseline was available for 382 (68%) patients, who displayed a median Wahlund score of 5.00 (IQR = 2.25–9.00). The Wahlund score at baseline as well as both models calculated (i.e. linear Wahlund Score and dichotomized with a cut-off), did not display any clinically relevant differences between high or low levels of RABs vs. comparator quartiles (Supplementary Tables 9 and 10).

## Discussion

We measured a panel of RABs directed against GPCRs including α1-abs, α2-abs, β1-abs, β2-abs, AT2-abs, ETB-abs and CXCR3-abs in patients with incident stroke. We observed more LAA strokes in patients with Q1 levels while more CE strokes were observed in patients with Q4 level RABs. Regarding functional outcome after one year, we observed worse mRS in patients with high levels (Q4) of alpha2-abs and ETB-abs vs. reference groups (Q1-Q3). Conversely, stroke patients with low levels (Q1) of AT2-abs, alpha2-abs, and beta1-abs had a higher hazard for a recurrent cardiovascular event or death within three years of follow-up, compared to the respective reference (Q2-Q4).

In a previous analysis of the PROSCIS-B cohort, other RABs, namely angiotensin II type 1 receptor (AT1R), endothelin‐1 type A receptor, complement factor‐3 and ‐5 receptors, vascular endothelial growth factor receptor‐1 and ‐2, vascular endothelial growth factor A, and factor B were analyzed in the context of functional outcome at one year and recurrent event risk over 3 years [28]. In line with this previous study, we found that for some RABs, namely α2- and ETB-abs, elevated levels were associated with worse functional outcome when compared to lower levels of the respective RAB in our study.

In this present study, we extended this analysis to low levels of RABs and also estimated the impact of low levels of RABs (Q1 vs. Q2-4) to account for a potential disbalance in both directions as previously referred to as the antibodiome [13]. We observed that indeed patients with low levels of α2-abs, β1-abs, and AT2-abs were at increased risk for a secondary event. These results align with findings reported in a cohort of patients with acute coronary syndrome: low levels (Q1) of β1-abs were linked to a higher risk of early recurrent MI and cardiovascular death within one year [27, 42]. While this was observed mainly in a subgroup of young ST elevation MI patients (≤ 60 years), our findings suggest that this link may be more generalizable, i.e., cross-disease and also in older patient populations.

A higher mortality, driven mainly by excess cardiac mortality, has been observed for high levels (Q4) of CXCR3-abs in the large population-based Gutenberg Health study, which comprised over 4000 healthy individuals [5]. In contrast, in our cohort high levels (Q4) of CXCR3-Abs were not associated with an increased risk of secondary events, possibly because we analyzed a combined endpoint rather than mortality alone. The apparent discrepancy may be explained by differences in study populations and endpoints, as our cohort consisted of patients with an incident stroke at study inclusion. A previous study has demonstrated that differential binding sites of GPCR targeting RABs exist between systemic sclerosis patients and healthy controls [22], implying that the underlying disease may impact the biology and antibody properties. The Gutenberg Health study has also reported a trend toward higher risk of major adverse cardiac events, including coronary artery disease, myocardial infarction, and cardiac death in patients with high levels of CXCR3-abs. In the LAA subgroup, we observed an almost threefold increase in recurrent event risk for high levels of CXCR3-abs. These observations may be explained by the fact that LAA stroke represents a cerebrovascular manifestation of systemic atherosclerosis and, therefore, shares key pathophysiological mechanisms with coronary artery disease and myocardial infarction.

Surprisingly, stroke etiology differed in patients with low and high RAB levels (Q1 vs Q4) for all RABs investigated, with the largest discrepancy observed for α1-abs for both CE and LAA strokes. Lower RAB levels may, therefore, reflect chronic inflammation or age-related declines of antibody levels. Moreover, some of the RABs, such as the AT2-abs, are moreover thought to exert vasodilative properties. [8] Lower levels of vasodilative RABs might foster a pro-atherogenic state or increased vasoconstriction, thereby promoting LAA. Moreover, LAA strokes show the highest recurrence rate among all stroke etiologies [43]. This could indicate possible associations of stroke etiology in the subgroup of low level RABs on cardiovascular secondary risk. However, causal mediation analysis did not support this hypothesis and we did not account for a higher number of recurrent events in the LAA subgroup, possibly due to limited sample size. In summary, none of our sensitivity analyses revealed an indication for a major impact of stroke etiology on our observed findings.

We observed more cardioembolic strokes in high levels of any RAB (Q4). Particularly beta-adrenergic RABs have been linked to atrial fibrillation [44], arrhythmias [20], and cardiomyocyte apoptosis leading to fibrosis and remodeling [44, 45]. Atrial fibrillation is the main risk factor for CE strokes and cardiac dilatation, fibrosis, and cardiomyopathy promote conduction abnormalities. Elevated beta-adrenergic RABs in CE could be causally linked to its pathology. There was an overall trend towards higher levels of inflammatory markers (e.g., IL-6, hsCRP) in patients with any RABs in Q4, which could be an additional contributor to clot formation (“thromboinflammation”) [46]. Patients with high RAB levels may consequently benefit from better secondary prevention strategies due to the higher number of CE strokes [47]. This phenomenon in turn may explain the non-apparent increase in secondary event risk in these patients. Our sensitivity analysis revealed the direct effect of high-level antibodies on the mRS category, but no mediation via CE stroke compared to other stroke etiologies.

We hypothesized that differences in CSVD burden may exist for patients with low vs. high levels of RABs, in particular for alpha adrenergic and AT2 RABs, as these antibodies had previously been associated with vasoconstrictive and pro-hypertensive properties. However, we did not find any associations of RAB levels with CSVD burden which may be explained by the high amount of missing data (imaging was not part of the primary study protocol of PROSCIS-B). Secondly, only a minor proportion of patients had relevant CSVD burden. Thirdly, no follow-up imaging was available to determine changes after the incident event due to blood–brain barrier changes.

We expected a link of high RAB levels with unfavorable cognitive outcomes as a potential consequence of an increased secondary CSVD burden. Previous studies have highlighted the role of anti-neuronal antibodies for cognitive outcome after ischemic stroke [48, 49]. However, we also did not record any trends pointing towards different cognitive outcomes, which underscores the need for further mechanistic research.

We expected medication use to potentially impact antibody levels, especially when pharmaceutical and antibody share target structures (such as beta blockers and β1/β2-abs). However, we rather observed medication intake to differ among RABs between Q1 and Q4 for almost all abs. This might reflect the different distribution of stroke etiologies rather than specific impacts of medication use on antibody levels.

### Strengths and limitations

Our data were derived from a large prospective cohort study, with a long follow-up of three years. However, due to low numbers of participants with cerebral bleedings or severe stroke events, we restricted our analysis to mild to moderate and ischemic stroke only. Therefore, our results may not be applicable to severe or non-ischemic cerebrovascular events.

We can only suspect that antibodies might have been preexisting as serum samples were collected on day 3–7; we neither have preexisting measurements to confirm nor multiple measurements to comment on the development of antibody levels over time and the influence of such dynamics on outcome. We only investigated antibodies of the IgG subclass and can, therefore, not comment on the potential impact or presence of other RABs of e.g. IgM or IgA subclass.

Regarding the antibody measurement, we are aware that the ELISA is very sensitive to changes due to environmental factors, such as temperature, and handling. While we tried to control this to the best of our abilities (standard protocols), we think that the rather wide range of quartiles might aid in correcting for some measurement imprecision inherent to the method of ELISA. Some samples (n = 12) were measured twice, independently, which showed good agreement when classed into quartiles. To ensure intra-assay precision, each sample was measured multiple times on the same plate. Additionally, serum samples carry the general risk of high background signal, antibody cross-reactivity, and interfering substances present in the sample, which could result in both over- and underestimation of antibody concentration.

Our main hypothesis concerning RABs included their possible effects on blood pressure as well as cardiomyopathy, especially for the adrenergic RABs. Some parameters that might have aided in entangling pathophysiological implications, such as longitudinal blood pressure data or echocardiographic imaging, were not routinely performed in all patients included. Therefore, we are lacking other outcome measures that may be of importance in the context of certain RABs and additionally cannot account for time-varying confounding.

We may not have eliminated confounding, which may be introduced by parameters not measured in the PROSCIS-B cohort, such as history of autoimmune disease or APO-E status or factors that have not been identified as relevant in this context yet. For the combined endpoint, we screened only in-house hospital records and patient reports, so events treated elsewhere may have gone undetected. However, we consider the impact of this rather small and non-differential.

As this study was designed as an exploratory analysis to investigate associations between different autoantibodies and outcomes, multiple statistical tests were conducted without a primary focus on a limited set of predefined hypotheses. This approach inherently increases the risk of Type I errors arising from the large number of tests performed. Accordingly, the observed associations should be interpreted with caution and regarded as hypothesis-generating. They require confirmation in independent validation cohorts.

## Conclusion

Our exploratory analyses indicate that RABs targeting GPCRs are linked to stroke characteristics, functional outcome, and recurrent cardiovascular risk in ischemic stroke patients. Higher RAB levels were associated with cardioembolic strokes, whereas lower levels were linked to large artery atherosclerosis. Additionally, high levels of α2- and ETB-antibodies correlated with worse functional outcomes, while low levels of α2-, β1-, and AT2-antibodies were associated with increased risk of recurrent cardiovascular events. Our exploratory findings suggest a possible role of some RABs in stroke pathophysiology, warranting further studies to investigate the underlying mechanisms.

## Supplementary Information

Below is the link to the electronic supplementary material.

Supplementary file1 (DOCX 4062 KB)
