# Supplementary material for: Autoantibodies targeting G protein-coupled receptors and clinical outcome after ischemic stroke (PROSCIS-B)
Source: J Neurol. 2026 Sep 8;273(10):581. doi: 10.1007/s00415-026-14103-6 (PMC13553654; doi:10.1007/s00415-026-14103-6)
Supplement: Supplementary file 1 [file 415_2026_14103_MOESM1_ESM.docx]

**Supplement:
Regulatory autoantibodies and clinical outcome after ischemic stroke (PROSCIS-B)**

Anna-Sophia Miesenberger ^1,2^, Friederike A. Arlt, MD ^2,3^, Leif Koschützke MD ^1,2^, Pimrapat Gebert PhD ^4^, Harald Heidecke PhD ^5^, Kai Schulze-Forster PhD ^5^, Rusan Ali Catar PhD ^6^, Guido Moll PhD ^6,7,8^, Christoph Harms MD ^1,2,9^, Anna Kufner MD, PhD^1,2^, Thomas Liman MD ^1,2,3^, Harald Prüß MD ^2, 3^, Matthias Endres MD ^1,2,3,9,10*^, Pia S. Sperber MD ^1,2,11*^

**these authors contributed equally*

*1 Center for Stroke Research Berlin (CSB), Berlin, Charité - Universitätsmedizin Berlin, Germany,*

*2 Department of Neurology and Experimental Neurology, Charité-Universitätsmedizin Berlin, Germany,*

*3 German Center for Neurodegenerative Diseases (DZNE), Berlin, Germany,*

*4 Institute of Biometry and Clinical Epidemiology, Charité-Universitätsmedizin Berlin, Germany,*

*5 CellTrend GmBH, Luckenwalde, Germany,*

*6 Department of Nephrology and Internal Intensive Care Medicine, Charité-Universitätsmedizin Berlin, Germany,*

*7 BIH Center for Regenerative Therapies (BCRT) Berlin, Germany,*

*8 Julius Wolff Institute for Musculoskeletal Research (JWI), Berlin, Germany,*

*9 German Centre for Cardiovascular Research (DZHK), Berlin, Germany*

*10 German Center for Mental Health (DZPG), Berlin, Germany*

*11 Experimental and Clinical Research Center, a Cooperation Between Max Delbrück Center for Molecular Medicine in the Helmholtz Association, Charité – Universitätsmedizin Berlin, Germany*

**Table of contents**

Supplementary Tables:

1. Baseline Characteristics of all defined Antibody Subgroups

2. Medication intake of all defined Antibody Subgroups

3. Dose-Response Analysis of Functional Outcome

4. Subgroup Analysis: Functional Outcome after 1 Year assessed by Modified Rankin Scale

5. Incidence Rates of Combined Endpoint (Stroke, Myocardial Infarction, Death of all cause)

6. Subgroup Analysis: Hazard Ratios for a Combined Endpoint over 3 Years

7. Dose-Response Analysis of Hazard Ratios for a Combined Endpoint over 3 years

8. TICS-m – linear mixed model

9. Wahlund Score - linear regression model

10. Wahlund Score - logistic regression model

Supplementary Figures:

1. Direct Acyclic Graphs – DAGitty Visualizations for confounder identification
2. Standardized Mean Differences - Visualizations Q1 vs Q2-4

3. Standardized Mean Differences - Visualizations Q4 vs Q1-3

4. mRS Grotta Bar Plots Q4 vs Q1-3 (remainder not displayed in full text)
5. mRS Grotta Bar Plots Q1 vs Q2-4

6. Causal Mediation Analysis of alpha2- and ETB-abs in Q4 on 1year mRS via Cardioembolic Stroke
7. Hazard Function Plots for Q1 vs. Q2-4 (remainder not displayed in full text)

8. Hazard Function Plots for Q4 vs. Q1-3

9. Causal Mediation Analysis of alpha2-, beta1- and AT2-abs in Q1 on log(Survival Time) via Large Artery Artherosclerosis Strokes

**Supplementary Table 1: Baseline Characteristics of all defined Antibody Subgroups**

|  | **Alpha 1** | | **Alpha 2** | | **Beta 1** | | **Beta 2** | | **Angiotensin 2** | | **Endothelin B** | | **CXCR3** | |
| --- | --- | --- | --- | --- | --- | --- | --- | --- | --- | --- | --- | --- | --- | --- |
|  | **Q1** | **Q4** | **Q1** | **Q4** | **Q1** | **Q4** | **Q1** | **Q4** | **Q1** | **Q4** | **Q1** | **Q4** | **Q1** | **Q4** |
| **Age** Mean (SD) | 70.2 (11.2) | 64.8 (13.3) | 68.0 (12.4) | 65.6 (12.8) | 68.9 (11.9) | 67.4 (12.9) | 68.9 (11.6) | 65.0 (13.9) | 67.4 (12.7) | 67.9 (12.1) | 69.0 (11.8) | 66.4 (12.8) | 69.8 (11.6) | 67.1 (13.1) |
| **Age Group** n (%) |  |  |  |  |  |  |  |  |  |  |  |  |  |  |
| <55 | 10 (7) | 33 (23) | 15 (11) | 28 (20) | 14 (10) | 23 (16) | 12 (9) | 33 (23) | 19 (14) | 25 (18) | 13 (9) | 27 (19) | 15 (11) | 24 (17) |
| 55-64 | 30 (21) | 31 (22) | 32 (23) | 39 (28) | 33 (23) | 31 (22) | 36 (26) | 32 (23) | 30 (21) | 32 (23) | 32 (23) | 33 (23) | 25 (18) | 35 (25) |
| 65-74 | 53 (38) | 40 (28) | 55 (39) | 34 (24) | 50 (36) | 39 (28) | 51 (36) | 36 (26) | 51 (36) | 35 (25) | 54 (38) | 38 (27) | 56 (40) | 36 (26) |
| >74 | 48 (34) | 37 (26) | 39 (28) | 40 (28) | 44 (31) | 48 (34) | 42 (30) | 40 (28) | 41 (29) | 49 (35) | 42 (30) | 43 (30) | 45 (32) | 46 (3) |
| **Sex** n (%) |  |  |  |  |  |  |  |  |  |  |  |  |  |  |
| Female | 56 (40) | 58 (41) | 57 (40) | 53 (38) | 57 (40) | 58 (41) | 57 (40) | 55 (39) | 62 (44) | 55 (39) | 60 (43) | 51 (36) | 56 (40) | 57 (40) |
| **Regular Alcohol Consumption** n (%) | 47 (33) | 47 (33) | 46 (33) | 48 (34) | 47 (33) | 40 (28) | 51 (36) | 46 (33) | 55 (39) | 48 (34) | 46 (33) | 44 (31) | 50 (36) | 40 (28) |
| **Active Smoking** | 35 (25) | 35 (25) | 39 (28) | 43 (31) | 33 (23) | 31 (22) | 38 (27) | 33 (23) | 32 (23) | 42 (30) | 36 (26) | 38 (27) | 31 (22) | 39 (28) |
| **Body Mass Index** Mean (SD) | 27.1 (4.62) | 27.7 (4.78) | 26.8 (4.71) | 27.6 (4.57) | 27.1 (5.14) | 27.8 (5.16) | 27.5 (5.00) | 27.8 (5.20) | 26.9 (5.02) | 27.6 (5.14) | 27.1 (5.11) | 27.8 (4.93) | 27.4 (5.36) | 27.5 (4.67) |
| **Systolic Blood Pressure (mmHg)** Mean (SD) | 140 (21.2) | 139 (21.8) | 140 (21.3) | 138 (22.1) | 142 (22.8) | 141 (22.7) | 141 (23.5) | 140 (22.0) | 142  (22.6) | 139  (22.6) | 142 (22.3) | 138 (23.0) | 141 (22.3) | 136 (22.3) |
| **Diastolic Blood Pressure (mmHg)** Mean (SD) | 77.6 (14.1) | 77.7 (15.3) | 77.6 (14.1) | 76.1 (14.9) | 78.0 (14.2) | 76.7 (15.0) | 78.4 (14.7) | 76.2 (15.5) | 77.7 (13.7) | 75.2 (14.9) | 77.9 (14.5) | 75.9 (15.6) | 76.9 (14.4) | 75.4 (14.4) |
| **Total Cholesterol** Mean (SD) | 188 (43.4) | 206 (43.5) | 191 (41.0) | 202 (48.5) | 195 (45.8) | 202 (44.3) | 192 (45.8) | 201 (44.1) | 197  (48.7) | 199  (44.3) | 193 (45.9) | 197 (42.5) | 195 (48.0) | 199 (43.0) |
| **Low Density Lipoprotein** Mean (SD) | 114 (38.2) | 127 (38.8) | 118 (38.2) | 125 (40.1) | 119 (39.4) | 124 (40.9) | 117 (39.1) | 121 (37.9) | 119  (39.9) | 121  (36.6) | 120 (39.8) | 120 (39.3) | 118 (40.3) | 121 (35.3) |
| **NIHSS at admission** |  |  |  |  |  |  |  |  |  |  |  |  |  |  |
| Median (Q1, Q3) | 2 (1, 5) | 3 (1, 4) | 2 (1, 5) | 2 (1, 4) | 2 (1, 4) | 3 (1, 5) | 2 (1, 5) | 3 (1, 4) | 2 (1, 5) | 3 (1, 4) | 2 (1, 5) | 3 (1, 4) | 2 (1, 5) | 3 (1, 5) |
| **NIHSS** n (%) |  |  |  |  |  |  |  |  |  |  |  |  |  |  |
| 0-4 | 105 (75) | 112 (79) | 98 (70) | 106 (75) | 108 (77) | 102 (72) | 101 (72) | 111 (79) | 99 (70) | 106 (75) | 105 (75) | 109 (77) | 103 (73) | 105 (75) |
| 5-15 | 36 (26) | 29 (21) | 43 (31) | 35 (25) | 33 (23) | 39 (28) | 40 (28) | 30 (21) | 42 (30) | 35 (25) | 36 (26) | 32 (23) | 38 (27) | 36 (26) |
| **Preexisting cv conditions**  n (%) |  |  |  |  |  |  |  |  |  |  |  |  |  |  |
| DMT2 | 35 (25) | 23 (16) | 34 (24) | 27 (19) | 33 (23) | 31 (22) | 33 (23) | 32 (23) | 33 (23) | 32 (23) | 34 (24) | 28 (20) | 28 (20) | 26 (18) |
| aHT | 94 (67) | 81 (57) | 91 (65) | 89 (63) | 94 (67) | 90 (64) | 94 (67) | 88 (62) | 90 (64) | 93 (66) | 99 (70) | 88 (62) | 102 (72) | 86 (61) |
| AFib | 35 (25) | 29 (21) | 32 (23) | 26 (18) | 38 (27) | 33 (23) | 31 (22) | 29 (21) | 27 (19) | 30 (21) | 31 (22) | 27 (19) | 34 (24) | 23 (16) |
| PAD | 14 (20) | 7 (5) | 10 (7) | 9 (6) | 9 (6) | 13 (9) | 13 (9) | 9 (6) | 7 (5) | 12 (9) | 11 (8) | 9 (6) | 8 (6) | 10 (7) |
| MI | 5 (4) | 4 (3) | 4 (3) | 3 (2) | 5 (4) | 4 (3) | 4 (3) | 5 (4) | 6 (4) | 3 (2) | 5 (4) | 3 (2) | 5 (4) | 2 (1) |
| AP | 27 (19) | 15 (11) | 26 (18) | 15 (11) | 25 (18) | 17 (12) | 24 (17) | 17 (12) | 26 (18) | 17 (12) | 20 (14) | 14 (10) | 27 (19) | 19 (14) |
| **high sensitivity CRP** Mean (SD) | 12.7 (22.8) | 12.0 (18.7) | 11.1 (20.0) | 16.9 (25.0) | 13.5 (23.7) | 16.8 (24.6) | 13.8 (23.9) | 15.8 (24.6) | 12.4 (22.0) | 16.3 (24.9) | 13.2 (23.1) | 16.0 (23.6) | 12.9 (22.8) | 15.1 (21.7) |
| **Treatment** n (%) |  |  |  |  |  |  |  |  |  |  |  |  |  |  |
| Thrombolysis | 31 (22) | 24 (17) | 41 (29) | 25 (18) | 29 (21) | 22 (16) | 35 (25) | 24 (17) | 32 (23) | 25 (18) | 35 (25) | 24 (17) | 35 (25) | 25 (18) |
| Thrombectomy | 4 (3) | 2 (1) | 2 (1) | 2 (1) | 1 (1) | 1 (1) | 2 (1) | 2 (1) | 1 (1) | 2 (1) | 1 (1) | 2 (1) | 2 (1) | 1 (1) |
| **Interleukin 6** |  |  |  |  |  |  |  |  |  |  |  |  |  |  |
| Mean (SD) | 10.9 (37.8) | 7.36 (12.2) | 10.7 (37.3) | 8.20 (12.0) | 10.2 (37.0) | 10.5 (17.3) | 7.12 (14.8) | 12.9 (44.1) | 7.59 (15.1) | 15.2 (69.5) | 10.3 (37.4) | 7.50 (11.0) | 10.0 (36.9) | 6.93 (10.9) |
| Interleukin 6 negative n (%) | 67 (48) | 57 (40) | 61 (43) | 52 (37) | 63 (45) | 44 (31) | 69 (49) | 49 (35) | 61 (43) | 49 (35) | 67 (48) | 49 (35) | 66 (47) | 53 (38) |
| Interleukin 6 positive n (%) | 68 (48) | 82 (58) | 76 (54) | 87 (62) | 71 (50) | 96 (68) | 67 (48) | 91 (65) | 74 (53) | 90 (64) | 69 (49) | 89 (63) | 69 (49) | 84 (60) |
| **Wahlund Score** |  |  |  |  |  |  |  |  |  |  |  |  |  |  |
| Mean (SD) | 6.89 (5.24) | 6.70 (5.87) | 6.57 (5.27) | 6.71 (5.75) | 6.99 (4.95) | 8.22 (6.63) | 7.43 (5.22) | 6.99 (6.13) | 7.12 (5.33) | 7.26 (5.80) | 7.09 (5.15) | 6.59 (5.94) | 6.96 (4.95) | 6.71 (5.99) |
| **Wahlund – Severity** n (%) |  |  |  |  |  |  |  |  |  |  |  |  |  |  |
| Mild (0-10) | 68 (48) | 73 (52) | 72 (51) | 74 (53) | 72 (51) | 60 (43) | 69 (49) | 72 (51) | 75 (53) | 67 (48) | 71 (50) | 68 (48) | 74 (53) | 69 (49) |
| Severe (≥10) | 27 (19) | 23 (16) | 27 (19) | 19 (14) | 28 (20) | 29 (21) | 31 (22) | 23 (16) | 30 (21) | 22 (16) | 26 (18) | 19 (14) | 27 (19) | 18 (13) |
| Missing | 46 (33) | 45 (32) | 42 (30) | 48 (34) | 41 (29) | 52 (37) | 41 (29) | 46 (33) | 36 (26) | 52 (37) | 44 (31) | 54 (38) | 40 (28) | 54 (38) |

Supplementary Table 1: Baseline characteristics of all subgroups of RABs (regulatory antibodies). Q1 – Quartile 1, Q4 – Quartile 4. Quartiles all comprise n = 141. Rounding might result in percentages not summing up to 100%. NIHSS; National Institute of Health Stroke Scale; cv, cardiovascular; DMT2, diabetes mellitus type 2; aHT, arterial hypertension; AFib, atrial fibrillation; PAD, peripheral arterial disease; MI, myocardial infarction; AP, angina pectoris; CRP, C-reactive protein; alpha1, alpha-1 adrenergic receptor antibodies; alpha2, alpha-2 adrenergic receptor antibodies; beta1, beta-1 adrenergic receptor antibodies; beta2, beta-2 adrenergic receptor antibodies; Angiotensin 2, angiotensin II receptor type 2 antibodies; Endothelin B, endothelin receptor type B antibodies; CXCR3d, chemokine receptor CXCR3 antibodies.

**Supplementary Table 2: Medication intake of all defined Antibody Subgroups**

|  | **Alpha 1** | | **Alpha 2** | | **Beta 1** | | **Beta 2** | | **Angiotensin 2** | | **Endothelin B** | | **CXCR3** | |  |
| --- | --- | --- | --- | --- | --- | --- | --- | --- | --- | --- | --- | --- | --- | --- | --- |
|  | **Q1** | **Q4** | **Q1** | **Q4** | **Q1** | **Q4** | **Q1** | **Q4** | **Q1** | **Q4** | **Q1** | **Q4** | **Q1** | **Q4** |  |
| **Beta-Blocker** |  |  |  |  |  |  |  |  |  |  |  |  |  |  |  |
| Baseline | 50 (35.0%) | 43 (30.5%) | 50 (35.0%) | 46 (32.4%) | 57 (40.1%) | 46 (32.4%) | 52 (36.1%) | 44 (31.2%) | 51 (35.7%) | 51 (35.9%) | 53 (37.1%) | 44 (31.0%) | 53 (37.1%) | 46 (32.4%) |  |
| 1y | 59 (41.3%) | 48 (34.0%) | 61 (42.7%) | 49 (34.5%) | 66 (46.5%) | 50 (35.2%) | 62 (43.1%) | 49 (34.8%) | 63 (44.1%) | 51 (35.9%) | 61 (42.7%) | 47 (33.1%) | 63 (44.1%) | 54 (38.0%) |  |
| 2y | 50 (35.0%) | 38 (27.0%) | 58 (40.6%) | 36 (25.4%) | 58 (40.8%) | 51 (35.9%) | 50 (34.7%) | 48 (34.0%) | 57 (39.9%) | 44 (31.0%) | 59 (41.3%) | 42 (29.6%) | 59 (41.3%) | 46 (32.4%) |  |
| 3y | 44 (30.8%) | 40 (28.4%) | 47 (32.9%) | 35 (24.6%) | 48 (33.8%) | 48 (33.8%) | 40 (27.8%) | 46 (32.6%) | 42 (29.4%) | 46 (32.4%) | 48 (33.6%) | 39 (27.5%) | 50 (35.0%) | 46 (32.4%) |  |
| **ACE/ARB** |  |  |  |  |  |  |  |  |  |  |  |  |  |  |  |
| Baseline | 52 (36.4%) | 43 (30.5%) | 54 (37.8%) | 44 (31.0%) | 58 (40.8%) | 48 (33.8%) | 47 (32.6%) | 49 (34.8%) | 54 (37.8%) | 45 (31.7%) | 58 (40.6%) | 47 (33.1%) | 57 (39.9%) | 48 (33.8%) |  |
| 1y | 69 (48.3%) | 57 (40.4%) | 64 (44.8%) | 65 (45.8%) | 72 (50.7%) | 60 (42.3%) | 72 (50.0%) | 62 (44.0%) | 72 (50.3%) | 69 (48.6%) | 72 (50.3%) | 65 (45.8%) | 70 (49.0%) | 69 (48.6%) |  |
| 2y | 61 (42.7%) | 53 (37.6%) | 64 (44.8%) | 56 (39.4%) | 69 (48.6%) | 62 (43.7%) | 65 (45.1%) | 62 (44.0%) | 67 (46.9%) | 65 (45.8%) | 70 (49.0%) | 64 (45.1%) | 62 (43.4%) | 64 (45.1%) |  |
| 3y | 55 (38.5%) | 50 (35.5%) | 55 (38.5%) | 47 (33.1%) | 53 (37.3%) | 55 (38.7%) | 53 (36.8%) | 55 (39.0%) | 54 (37.8%) | 52 (36.6%) | 61 (42.7%) | 54 (38.0%) | 56 (39.2%) | 54 (38.0%) |  |
| **Antiplatelet** |  |  |  |  |  |  |  |  |  |  |  |  |  |  |  |
| Baseline | 46 (32.2%) | 33 (23.4%) | 46 (32.2%) | 30 (21.1%) | 42 (29.6%) | 38 (26.8%) | 44 (30.6%) | 40 (28.4%) | 45 (31.5%) | 41 (28.9%) | 47 (32.9%) | 38 (26.8%) | 43 (30.1%) | 37 (26.1%) |  |
| 1y | 63 (44.1%) | 71 (50.4%) | 52 (36.4%) | 76 (53.5%) | 58 (40.8%) | 72 (50.7%) | 66 (45.8%) | 73 (51.8%) | 61 (42.7%) | 71 (50.0%) | 56 (39.2%) | 76 (53.5%) | 64 (44.8%) | 79 (55.6%) |  |
| 2y | 72 (50.3%) | 61 (43.3%) | 66 (46.2%) | 59 (41.5%) | 63 (44.4%) | 64 (45.1%) | 75 (52.1%) | 68 (48.2%) | 70 (49.0%) | 63 (44.4%) | 67 (46.9%) | 64 (45.1%) | 65 (45.5%) | 66 (46.5%) |  |
| 3y | 62 (43.4%) | 53 (37.6%) | 56 (39.2%) | 55 (38.7%) | 54 (38.0%) | 54 (38.0%) | 65 (45.1%) | 56 (39.7%) | 57 (39.9%) | 57 (40.1%) | 55 (38.5%) | 58 (40.8%) | 58 (40.6%) | 58 (40.8%) |  |
| **Anticoagulation** |  |  |  |  |  |  |  |  |  |  |  |  |  |  |  |
| Baseline | 7 (4.9%) | 8 (5.7%) | 5 (3.5%) | 13 (9.2%) | 8 (5.6%) | 13 (9.2%) | 5 (3.5%) | 8 (5.7%) | 6 (4.2%) | 13 (9.2%) | 7 (4.9%) | 14 (9.9%) | 9 (6.3%) | 9 (6.3%) |  |
| 1y | 26 (18.2%) | 27 (19.1%) | 31 (21.7%) | 23 (16.2%) | 32 (22.5%) | 26 (18.3%) | 24 (16.7%) | 23 (16.3%) | 28 (19.6%) | 28 (19.7%) | 28 (19.6%) | 25 (17.6%) | 29 (20.3%) | 26 (18.3%) |  |
| 2y | 25 (17.5%) | 25 (17.7%) | 28 (19.6%) | 21 (14.8%) | 31 (21.8%) | 30 (21.1%) | 20 (13.9%) | 27 (19.1%) | 25 (17.5%) | 30 (21.1%) | 27 (18.9%) | 27 (19.0%) | 30 (21.0%) | 23 (16.2%) |  |
| 3y | 21 (14.7%) | 24 (17.0%) | 27 (18.9%) | 21 (14.8%) | 25 (17.6%) | 29 (20.4%) | 16 (11.1%) | 28 (19.9%) | 18 (12.6%) | 26 (18.3%) | 23 (16.1%) | 24 (16.9%) | 28 (19.6%) | 25 (17.6%) |  |
| **Lipid Lowering** |  |  |  |  |  |  |  |  |  |  |  |  |  |  |  |
| Baseline | 38 (26.6%) | 18 (12.8%) | 39 (27.3%) | 16 (11.3%) | 38 (26.8%) | 19 (13.4%) | 39 (27.1%) | 22 (15.6%) | 34 (23.8%) | 18 (12.7%) | 37 (25.9%) | 19 (13.4%) | 37 (25.9%) | 21 (14.8%) |  |
| 1y | 80 (55.9%) | 64 (45.4%) | 82 (57.3%) | 70 (49.3%) | 86 (60.6%) | 74 (52.1%) | 86 (59.7%) | 70 (49.6%) | 87 (60.8%) | 73 (51.4%) | 81 (56.6%) | 72 (50.7%) | 92 (64.3%) | 74 (52.1%) |  |
| 2y | 67 (46.9%) | 59 (41.8%) | 65 (45.5%) | 58 (40.8%) | 69 (48.6%) | 63 (44.4%) | 65 (45.1%) | 64 (45.4%) | 68 (47.6%) | 62 (43.7%) | 68 (47.6%) | 64 (45.1%) | 76 (53.1%) | 62 (43.7%) |  |
| 3y | 56 (39.2%) | 50 (35.5%) | 58 (40.6%) | 47 (33.1%) | 56 (39.4%) | 53 (37.3%) | 55 (38.2%) | 53 (37.6%) | 53 (37.1%) | 51 (35.9%) | 55 (38.5%) | 53 (37.3%) | 58 (40.6%) | 51 (35.9%) |  |

Supplementary Table 2: Medication intake at baseline as well as 1, 2 and 3 year follow up for all RABs. Q1 – Quartile 1, Q4 – Quartile 4. Quartiles all comprise n = 141. Rounding might result in percentages not summing up to 100%. alpha1, alpha-1 adrenergic receptor antibodies; alpha2, alpha-2 adrenergic receptor antibodies; beta1, beta-1 adrenergic receptor antibodies; beta2, beta-2 adrenergic receptor antibodies; Angiotensin 2, angiotensin II receptor type 2 antibodies; Endothelin B, endothelin receptor type B antibodies; CXCR3d, chemokine receptor CXCR3 antibodies.

**Supplementary Table 3: *Dose-Response Analysis of Functional Outcome***

|  | **Alpha1**  *OR (95%CI)* | **Alpha2**  *OR (95%CI)* | **Beta1**  *OR (95%CI)* | **Beta2**  *OR (95%CI)* | **Angiotensin2**  *OR (95%CI)* | **Endothelin B**  *OR (95%CI)* | **CXCR3**  *OR (95%CI)* |
| --- | --- | --- | --- | --- | --- | --- | --- |
| Q1 (Ref.) | 1 | 1 | 1 | 1 | 1 | 1 | 1 |
| Q2 | 1.38 (0.87, 2.21) | 1.00 (0.63, 1.61) | 1.70 (1.07, 2.71) | 1.23 (0.77, 1.96) | 0.72 (0.45, 1.16) | 1.05 (0.66, 1.67) | 1.17 (0.73, 1.86) |
| Q3 | 1.27 (0.80, 2.04) | 1.17 (0.73, 1.87) | 1.19 (0.75, 1.90) | 1.08 (0.68, 1.71) | 0.96 (0.60, 1.53) | 1.09 (0.68, 1.74) | 1.40 (0.87, 2.24) |
| Q4 | 1.05 (0.65, 1.69) | 1.60 (1.00, 2.57) | 1.19 (0.74, 1.91) | 1.01 (0.63, 1.63) | 1.20 (0.75, 1.91) | 1.58 (1.00, 2.54) | 1.42 (0.89, 2.27) |

Supplementary Table 3: Functional outcome one year after first ischemic stroke assessed by mRS (modified Rankin Scale) adjusted for age, sex, and systemic inflammation (hsCRP) using a proportional odds logistic regression model. OR Odds Ratio from the proportional odds logistic regression; 95%-CI, 95% Confidence interval. Dose-Response Analysis using Quartile (Q) 1 as a Reference (Ref.).

**Supplementary Table 4: *Functional Outcome after 1 Year assessed by Modified Rankin Scale***

| **Antibody target** | | **LAA** | | **CE** | |
| --- | --- | --- | --- | --- | --- |
|  |  | *OR* | *95%CI* | *OR* | *95%CI* |
| Alpha 1 | *Q1 vs Q2-4* | 0.86 | (0.44 – 1.66) | 0.86 | (0.35 – 2.08) |
|  | *Q4 vs Q1-3* | 1.12 | (0.48 – 2.61) | 1.22 | (0.58 – 2.60) |
| Alpha 2 | *Q1 vs Q2-4* | 0.55 | (0.27 – 1.12) | 0.79 | (0.36 – 1.70) |
|  | *Q4 vs Q1-3* | 1.39 | (0.60 – 3.19) | 1.31 | (0.61 – 2.82) |
| Beta 1 | *Q1 vs Q2-4* | 0.68 | (0.31 – 1.45) | 0.56 | (0.29 – 1.17) |
|  | *Q4 vs Q1-3* | 0.86 | (0.40 – 1.85) | 1.04 | (0.48 – 2.25) |
| Beta 2 | *Q1 vs Q2-4* | 1.26 | (0.65 – 2.46) | 0.78 | (0.34 – 1.76) |
|  | *Q4 vs Q1-3* | 0.80 | (0.35 – 1.80) | 1.58 | (0.72 – 3.49) |
| AT2 | *Q1 vs Q2-4* | 0.85 | (0.42 – 1.71) | 1.18 | (0.54 – 2.58) |
|  | *Q4 vs Q1-3* | 0.64 | (0.28 – 1.42) | 1.63 | (0.77 – 3.46) |
| ETB | *Q1 vs Q2-4* | 0.93 | (0.46 – 1.90) | 0.71 | (0.32 – 1.59) |
|  | *Q4 vs Q1-3* | 0.89 | (0.37 – 2.11) | 1.70 | (0.80 – 3.60) |
| CXCR3 | *Q1 vs Q2-4* | 0.96 | (0.49 – 1.86) | 0.61 | (0.26 – 1.40) |
|  | *Q4 vs Q1-3* | 1.23 | (0.52– 2.93) | 1.22 | (0.57 – 2.62) |

Supplementary Table 4: Functional outcome one year after first ischemic stroke assessed by mRS (modified Rankin Scale) adjusted for age, sex, and systemic inflammation (hsCRP) within the subgroups of large artery atherosclerosis (LAA) and cardioembolic (CE) stroke. OR Odds Ratio from the proportional odds logistic regression; 95%-CI, 95% Confidence interval. alpha1-abs, alpha-1 adrenergic receptor antibodies; alpha2-abs, alpha-2 adrenergic receptor antibodies; beta1-abs, beta-1 adrenergic receptor antibodies; beta2-abs, beta-2 adrenergic receptor antibodies; AT2-abs, angiotensin II receptor type 2 antibodies; ETB-abs, endothelin receptor type B antibodies; CXCR3-abs, chemokine receptor CXCR3 antibodies. Q, Quartile.

**Supplementary Table 5: *Incidence Rates of Combined Endpoint (Stroke, Myocardial Infarction, Death of all cause)***

| **Antibody target** | | **Events** | **Person Years** | **Incidence Rate (95%CI)** *per 100 person years* |
| --- | --- | --- | --- | --- |
| Alpha 1 | *Q1* | 28 | 346 | 8.09 (5.38 - 11.7) |
|  | *Q4* | 17 | 354 | 4.80 (2.79 - 7.68) |
| Alpha 2 | *Q1* | 33 | 342 | 9.65 (6.64 - 13.6) |
|  | *Q4* | 22 | 352 | 6.24 (3.91 - 9.45) |
| Beta 1 | *Q1* | 33 | 347 | 9.50 (6.54 - 13.3) |
|  | *Q4* | 21 | 360 | 5.83 (3.61 - 8.91) |
| Beta 2 | *Q1* | 31 | 339 | 9.14 (6.21 - 13.0) |
|  | *Q4* | 20 | 362 | 5.53 (3.38 - 8.54) |
| AT2 | *Q1* | 34 | 350 | 9.71 (6.72 - 13.6) |
|  | *Q4* | 18 | 364 | 4.94 (2.93 - 7.81) |
| ETB | *Q1* | 31 | 356 | 8.70 (5.91 - 12.4) |
|  | *Q4* | 20 | 362 | 5.53 (3.38 - 8.54) |
| CXCR3 | *Q1* | 27 | 359 | 7.52 (4.96 - 10.9) |
|  | *Q4* | 23 | 354 | 6.50 (4.12 - 9.75) |

Supplementary Table 5: Events, person years and incidence rate per 100 person years for a combined endpoint (stroke, myocardial infraction, death of all cause). alpha1, alpha-1 adrenergic receptor antibodies; alpha2, alpha-2 adrenergic receptor antibodies; beta1, beta-1 adrenergic receptor antibodies; beta2, beta-2 adrenergic receptor antibodies; AT2, angiotensin II receptor type 2 antibodies; ETB, endothelin receptor type B antibodies; CXCR3, chemokine receptor CXCR3 antibodies. Q, Quartile; CI, Confidence interval.

**Supplementary Table 6: *Hazard Ratios for a Combined Endpoint over 3 years***

| **Antibody target** | | **LAA** | | **CE** | |
| --- | --- | --- | --- | --- | --- |
|  |  | *HR* | *95%CI* | *HR* | *95%CI* |
| Alpha 1 | *Q1 vs Q2-4* | 0.40 | (0.14 – 1.19) | 1.18 | (0.48 – 2.89) |
|  | *Q4 vs Q1-3* | 1.19 | (0.43 – 3.28) | 0.60 | (0.22 – 1.64) |
| Alpha 2 | *Q1 vs Q2-4* | 0.97 | (0.40 – 2.39) | 2.34 | (1.05 – 5.24) |
|  | *Q4 vs Q1-3* | 1.33 | (0.51 – 3.53) | 1.24 | (0.50 – 3.09) |
| Beta 1 | *Q1 vs Q2-4* | 0.82 | (0.32 – 2.15) | 1.41 | (0.62 – 3.23) |
|  | *Q4 vs Q1-3* | 1.20 | (0.49 – 2.93) | 0.80 | (0.33 – 1.95) |
| Beta 2 | *Q1 vs Q2-4* | 0.81 | (0.33 -2.00) | 2.31 | (1.02 – 5.23) |
|  | *Q4 vs Q1-3* | 1.06 | (0.41 – 2.70) | 0.89 | (0.35 – 2.27) |
| AT2 | *Q1 vs Q2-4* | 1.26 | (0.53 – 2.99) | 1.52 | (0.65 – 3.53) |
|  | *Q4 vs Q1-3* | 0.60 | (0.20 – 1.79) | 0.84 | (0.35 – 2.04) |
| ETB | *Q1 vs Q2-4* | 0.76 | (0.29 – 1.98) | 2.30 | (1.04 – 5.09) |
|  | *Q4 vs Q1-3* | 0.88 | (0.29 – 2.64) | 1.09 | (0.47 – 2.56) |
| CXCR3 | *Q1 vs Q2-4* | 0.67 | (0.26 – 1.74) | 1.27 | (0.55 – 2.96) |
|  | *Q4 vs Q1-3* | 2.69 | (1.15 – 6.32) | 0.99 | (0.39 – 2.53) |

Supplementary Table 6: Hazard ratios of high and low antibody-levels for the respective antibody adjusted for age, sex, and systemic inflammation (hsCRP) within the subgroups of large artery atherosclerosis (LAA) and cardioembolic (CE) stroke. HR, hazard ratio; 95% CI, 95% confidence interval; Q1, measurements within the first quartile of antibody levels. alpha1-abs, alpha-1 adrenergic receptor antibodies; alpha2-abs, alpha-2 adrenergic receptor antibodies; beta1-abs, beta-1 adrenergic receptor antibodies; beta2-abs, beta-2 adrenergic receptor antibodies; AT2-abs, angiotensin II receptor type 2 antibodies; ETB-abs, endothelin receptor type B antibodies; CXCR3-abs, chemokine receptor CXCR3 antibodies. Q, Quartile. LAA, large artery atherosclerosis; CE, cardioembolic stroke.

**Supplementary Table 7: *Dose-Response Analysis of Hazard Ratios for a Combined Endpoint over 3 years***

|  | **Alpha1**  *HR (95%CI)* | **Alpha2**  *HR (95%CI)* | **Beta1**  *HR (95%CI)* | **Beta2**  *HR (95%CI)* | **Angiotensin2**  *HR (95%CI)* | **Endothelin B**  *HR (95%CI)* | **CXCR3**  *HR (95%CI)* |
| --- | --- | --- | --- | --- | --- | --- | --- |
| Q1 (Ref.) | 1 | 1 | 1 | 1 | 1 | 1 | 1 |
| Q2 | 1.14 (0.67, 1.95) | 0.59 (0.34, 1.01) | 0.53 (0.28, 0.97) | 0.56 (0.30, 1.04) | 0.57 (0.33, 1.00) | 0.84 (0.49, 1.45) | 1.01 (0.58, 1.76) |
| Q3 | 0.73 (0.41, 1.33) | 0.42 (0.23, 0.79) | 0.74 (0.43, 1.28) | 0.80 (0.47, 1.35) | 0.57 (0.33, 1.00) | 0.57 (0.31, 1.02) | 0.75 (0.41, 1.39) |
| Q4 | 0.74 (0.40, 1.36) | 0.69 (0.40, 1.19) | 0.62 (0.36, 1.08) | 0.68 (0.38, 1.20) | 0.47 (0.27, 0.84) | 0.67 (0.38, 1.18) | 0.93 (0.53, 1.62) |

Supplementary Table 7: Hazard ratio for a combined endpoint (stroke, myocardial infarction, all-cause death) over 3 years adjusted for age, sex, and systemic inflammation (hsCRP) using a Cox proportional hazard model. HR Hazard Ratio from Cox proportional hazard model; 95%-CI, 95% Confidence interval. Dose-Response Analysis using Quartile (Q) 1 as a Reference (Ref.).

**Supplementary Table 8: Cognitive Outcome assessed by TICS-m (telephone interview for cognitive status modified) with a linear mixed model over 3 Years**

| **Antibody target** | | **Model 1** | | **Model 2** | | **Model 3** | |
| --- | --- | --- | --- | --- | --- | --- | --- |
|  |  | *β* | *(95%CI)* | *β* | *(95%CI)* | *β* | *(95%CI)* |
| Alpha 1 | *Q1 vs Q2-4* | 0.02 | (-1.06 ­– 1.10) | 0.53 | (-0.50 – 1.56) | 0.54 | (-0.49 – 1.56) |
|  | *Q4 vs Q1-3* | 1.19 | (0.10 – 2.28) | 0.83 | (-0.21 – 1.87) | 0.80 | (-0.24 – 1.83) |
| Alpha 2 | *Q1 vs Q2-4* | 0.20 | (-0.88 – 1.28) | 0.35 | (-0.68 – 1.37) | 0.27 | (-0.75 – 1.29) |
|  | *Q4 vs Q1-3* | -0.86 | (-1.96 – 0.25) | -1.25 | (-3.00 – -0.21) | -1.07 | (-2.13 – -0.02) |
| Beta 1 | *Q1 vs Q2-4* | -0.28 | (-1.37 – 0.81) | -0.03 | (-1.07 – 1.01) | -0.06 | (-1.09 – 0.97) |
|  | *Q4 vs Q1-3* | -1.03 | (-2.12 – 0.06) | -0.97 | (-2.01 – 0.06) | -0.74 | (-1.78 – 0.30) |
| Beta 2 | *Q1 vs Q2-4* | -0.60 | (-1.68 – 0.47) | -0.27 | (-1.30 – 0.75) | -0.25 | (-1.27 – 0.77) |
|  | *Q4 vs Q1-3* | -0.03 | (-1.12 – 1.06) | -0.27 | (-1.31 – 0.76) | -0.01 | (-1.05 – 1.02) |
| AT2 | *Q1 vs Q2-4* | 0.02 | (-1.05 – 1.10) | -0.10 | (-0.34 – 0.74) | -0.11 | (-1.12 – 0.91) |
|  | *Q4 vs Q1-3* | -1.21 | (-2.29 – -0.13) | -1.16 | (-2.19 – -0.13) | -1.00 | (-2.03 – 0.02) |
| ETB | *Q1 vs Q2-4* | -0.10 | (-1.17 – 0.97) | 0.11 | (-0.91 – 1.13) | 0.12 | (-0.89 – 1.13) |
|  | *Q4 vs Q1-3* | -0.68 | (-1.75 – 0.39) | -0.75 | (-1.76 – 0.27) | -0.61 | (-1.62 – 0.41) |
| CXCR3 | *Q1 vs Q2-4* | 0.39 | (-0.51 – 1.03) | 0.80 | (-0.21 – 1.80) | 0.77 | (-0.23 – 1.77) |
|  | *Q4 vs Q1-3* | -0.23 | (-1.31 – 0.86) | -0.28 | (-1.31 – 0.75) | -0.17 | (-1.19 – 0.85) |

Supplementary Table 8: Cognitive outcome assessed by TICS-m (telephone interview for cognitive status modified) with a linear mixed model over 3 years. AT2, Angiotensin II Receptor Type 2; ETB, Endothelin B; β, regression coefficient; 95%CI, 95% Confidence Interval; Model 1, unadjusted crude model; Model 2, adjusted for age and sex, Model 3, adjusted for age, sex and systemic inflammation (hsCRP). alpha1, alpha-1 adrenergic receptor antibodies; alpha2, alpha-2 adrenergic receptor antibodies; beta1, beta-1 adrenergic receptor antibodies; beta2, beta-2 adrenergic receptor antibodies; AT2, angiotensin II receptor type 2 antibodies; ETB, endothelin receptor type B antibodies; CXCR3, chemokine receptor CXCR3 antibodies. Q, Quartile.

**Supplementary Table 9: Wahlund Score at Baseline - linear regression model**

| **Antibody target** | | **Model 1** | | **Model 2** | | **Model 3** | |
| --- | --- | --- | --- | --- | --- | --- | --- |
|  |  | *β* | *(95%CI)* | *β* | *(95%CI)* | *β* | *(95%CI)* |
| Alpha 1 | *Q1 vs Q2-4* | 0.44 | (0.82 – 1.70) | -0.43 | (-1.58 – 0.73) | -0.40 | (-1.56 – 0.75) |
|  | *Q4 vs Q1-3* | 0.18 | (-1.08 – 1.44) | 0.76 | (-0.38 – 1.90) | 0.77 | (-0.37 – 1.91) |
| Alpha 2 | *Q1 vs Q2-4* | 0.00 | (-1.25 – 1.25) | -0.51 | (-1.64 – 0.61) | -0.45 | (-1.58 – 0.68) |
|  | *Q4 vs Q1-3* | 0.19 | (-1.08 – 1.46) | 0.56 | (-0.59 – 1.71) | 0.47 | (-0.70 – 1.63) |
| Beta 1 | *Q1 vs Q2-4* | 0.57 | (-0.67 – 1.82) | 0.04 | (-1.08 – 1.17) | 0.08 | (-1.05 – 1.21) |
|  | *Q4 vs Q1-3* | 2.16 | (0.89 – 3.44) | 1.98 | (0.83 – 3.12) | 1.94 | (0.78 – 3.09) |
| Beta 2 | *Q1 vs Q2-4* | 1.17 | (-0.06 – 2.41) | 0.50 | (-0.63 – 1.62) | 0.51 | (-0.62 – 1.63) |
|  | *Q4 vs Q1-3* | 0.56 | (-0.70 – 1.83) | 1.26 | (0.12 – 2.40) | 1.24 | (0.09 – 2.38) |
| AT2 | *Q1 vs Q2-4* | 0.77 | (-0.45 – 1.99) | 0.39 | (-0.72 – 1.50) | 0.43 | (-0.68 – 1.54) |
|  | *Q4 vs Q1-3* | 0.90 | (-0.38 – 2.19) | 0.78 | (-0.38 – 1.94) | 0.71 | (-0.46 – 1.88) |
| ETB | *Q1 vs Q2-4* | 0.71 | (-0.55 – 1.96) | -0.18 | (-1.33 – 0.96) | -0.17 | (-1.31 – 0.98) |
|  | *Q4 vs Q1-3* | 0.03 | (-1.27 – 1.33) | 0.40 | (-0.77 – 1.58) | 0.31 | (-0.87 – 1.50) |
| CXCR3 | *Q1 vs Q2-4* | 0.54 | (-0.70 – 1.77) | -0.35 | (-1.48 – 0.78) | -0.35 | (-1.48 – 0.78) |
|  | *Q4 vs Q1-3* | 0.19 | (-1.11 – 1.49) | 0.22 | (-0.96 – 1.39) | 0.28 | (-1.00 – 1.35) |

Supplementary Table 9: Wahlund Score linear model. AT2, Angiotensin II Receptor Type 2; ETB, Endothelin B; β, regression coefficient; 95%CI, 95% Confidence Interval; Model 1, unadjusted crude model; Model 2, adjusted for age and sex, Model 3, adjusted for age, sex and systemic inflammation (hsCRP). alpha1, alpha-1 adrenergic receptor antibodies; alpha2, alpha-2 adrenergic receptor antibodies; beta1, beta-1 adrenergic receptor antibodies; beta2, beta-2 adrenergic receptor antibodies; AT2, angiotensin II receptor type 2 antibodies; ETB, endothelin receptor type B antibodies; CXCR3, chemokine receptor CXCR3 antibodies. Q, Quartile.

**Supplementary Table 10: Wahlund Score at Baseline - logistic regression model**

| **Antibody target** | | **Model 1** | | **Model 2** | | **Model 3** | |
| --- | --- | --- | --- | --- | --- | --- | --- |
|  |  | *β* | *(95%CI)* | *β* | *(95%CI)* | *β* | *(95%CI)* |
| Alpha 1 | *Q1 vs Q2-4* | 0.28 | (-0.25 – 0.80) | 0.05 | (-0.51 – 0.60) | 0.05 | (-0.52 – 0.60) |
|  | *Q4 vs Q1-3* | -0.02 | (-0.58 – 0.50) | 0.15 | (-0.45 – 0.72) | 0.15 | (-0.45 – 0.72) |
| Alpha 2 | *Q1 vs Q2-4* | 0.21 | (-0.32 – 0.72) | 0.06 | (-0.51 – 0.61) | 0.06 | (-0.51 – 0.61) |
|  | *Q4 vs Q1-3* | -0.29 | (-0.88 – 0.26) | -0.18 | (-0.81 – 0.42) | -0.17 | (-0.80 – 0.43) |
| Beta 1 | *Q1 vs Q2-4* | 0.26 | (-0.27 – 0.77) | 0.13 | (-0.43 – 0.68) | 0.13 | (-0.43 – 0.67) |
|  | *Q4 vs Q1-3* | 0.55 | (0.02- –.07) | 0.51 | (-0.06 – 1.07) | 0.51 | (-0.06 – 1.07) |
| Beta 2 | *Q1 vs Q2-4* | 0.37 | (-0.05 – 0.97) | 0.31 | (-0.23 – 0.85) | 0.31 | (-0.24 – 0.85) |
|  | *Q4 vs Q1-3* | -0.01 | (-0.57 – 0.52) | 0.20 | (-0.40- –.78) | 0.20 | (-0.35 – 0.74) |
| AT2 | *Q1 vs Q2-4* | 0.31 | (-0.21 – 0.81) | 0.21 | (-0.34 – 0.74) | 0.20 | (-0.35 – 0.74) |
|  | *Q4 vs Q1-3* | 0.03 | (-0.54 – 0.57) | 0.02 | (-0.58 – 0.60) | 0.02 | (-0.59 – 0.60) |
| ETB | *Q1 vs Q2-4* | 0.18 | (-0.36 – 0.69) | -0.12 | (-0.70 – 0.43) | -0.13 | (-0.70 – 0.43) |
|  | *Q4 vs Q1-3* | -0.18 | (-0.77 – 0.38) | -0.05 | (-0.68 – 0.54) | -0.04 | (-0.67 – 0.56) |
| CXCR3 | *Q1 vs Q2-4* | 0.17 | (-0.36 – 0.68) | -0.11 | (-0.68 – 0.44) | -0.11 | (-0.68 – 0.43) |
|  | *Q4 vs Q1-3* | -0.27 | (-0.87 – 0.30) | -0.32 | (-0.97 – 0.28) | -0.33 | (-0.97 – 0.28) |

Supplementary Table 10: Wahlund Score logistic Regression for dichotomized outcome of Wahlund scores <10 vs >= 10. AT2, Angiotensin II Receptor Type 2; ETB, Endothelin B; β, regression coefficient; 95%CI, 95% Confidence Interval; Model 1, unadjusted crude model; Model 2, adjusted for age and sex, Model 3, adjusted for age, sex, and systemic inflammation (hsCRP). alpha1, alpha-1 adrenergic receptor antibodies; alpha2, alpha-2 adrenergic receptor antibodies; beta1, beta-1 adrenergic receptor antibodies; beta2, beta-2 adrenergic receptor antibodies; AT2, angiotensin II receptor type 2 antibodies; ETB, endothelin receptor type B antibodies; CXCR3, chemokine receptor CXCR3 antibodies. Q, Quartile.

**Supplementary Figure 1: Direct Acyclic Graphs (DAGs) visualizing Confounder Identification**

Supplementary Figure 1: direct acyclic graphs (DAGs) visualizing confounder identification process using DAGitty. A Confounder selection for the effect estimation of RABs on functional outcome (mRS). B Confounder selection for cognitive outcome (TICS-m). C Confounder selection for combined endpoint (recurrent stroke, MI, all cause death).

**Supplementary Figure 2: Standardized Mean Difference for Antibodies in Q1 vs Q2-4**

Supplementary Figure 2: Standardized mean difference (SMD) for Quartile (Q)1 vs. Q2-4 for all antibodies. Red vertical line at 0.1 represents commonly used cut-off for significance. BP, blood pressure; IL-6, Interleukin 6; NIHSS, National Institute of Health Stroke Scale; hsCRP, high sensitivity C-reactive Protein; LDL, low density lipoprotein. A alpha1. B alpha2. C beta1. D beta2. E Angiotensin II Receptor Type 2. F Endothelin B. G CXCR3.

**Supplementary Figure 3: Standardized Mean Difference for Antibodies in Q4 vs Q1-3**

 Supplementary Figure 3: Standardized mean difference (SMD) for Quartile (Q)4 vs. Q1-3 for all antibodies. Red vertical line at 0.1 represents commonly used cut-off for significance. BP, blood pressure; IL-6, Interleukin 6; NIHSS, National Institute of Health Stroke Scale; hsCRP, high sensitivity C-reactive Protein; LDL, low density lipoprotein. A alpha1. B alpha2. C beta1. D beta2. E Angiotensin 2. F Endothelin B. G CXCR3.

**Supplementary Figure 4: mRS at one Year Q1 vs. Q2-4**

Supplementary Figure4: Grotta bar plots visualizing difference functional outcome (mRS, modified Rankin Scale) after 1 year. Comparison of Quartile (Q)1 vs. Q2-4. mRS categories 5 + 6 were collapsed into one category. A alpha1. B alpha2. C beta1. D beta2. E Angiotensin 2. F Endothelin B. G CXCR3.

**Supplementary Figure 5: mRS at one Year Q4 vs. Q1-3**

Supplementary Figure 5: Grotta bar plots visualizing difference functional outcome (mRS, modified Rankin Scale) after 1 year. Comparison of Quartile (Q)4 vs. Q1-3. mRS categories 5 + 6 were collapsed into one category. A alpha1. B beta1. C beta2. D Angiotensin 2. E CXCR3.

**Supplementary Figure 6: Causal Mediation Analysis of alpha2- and ETB-abs in Q4 on one Year mRS via Cardioembolic Stroke**

Supplementary Figure 6: Causal mediation analysis of alpha2 adrenergic and Endothelin B (ETB) receptor antibodies on functional outcome via potential mediation through cardioembolic strokes. ACME = average causal mediation effect, ADE = average direct effect. mRS = modified rankin scale. Effects for mRS categories are sorted top to bottom as follows: 0, 1, 2, 3, 4 and 5+6 for ACME, ADE and total effect.

**Supplementary Figure 7: Cumulative Hazard Functions for Combined Endpoint over Three Years (Q1 vs. Q2-4)**

Supplementary Figure 7: Cumulative hazard functions over 3 years after incident ischemic stroke for a combined endpoint (recurrent stroke, myocardial infarction, all-cause death). Vertical lines on the graph represent an event. Comparisons shown for Quartile (Q)1 vs. Q2-4. A alpha1; B beta2; C Endothelin B; D CXCR3.

**Supplementary Figure 8: Cumulative Hazard Functions for Combined Endpoint over Three Years (Q4 vs. Q1-3)**

Supplementary Figure 8: Cumulative hazard functions over 3 years after incident ischemic stroke for a combined endpoint (recurrent stroke, myocardial infarction, all-cause death). Vertical lines on the graph represent an event. Comparisons shown for Quartile (Q)4 vs. Q1-3. A alpha1. B alpha2. C beta1. D beta2. E Angiotensin 2. F Endothelin B. G CXCR3.

**Supplementary Figure 9: Causal Mediation Analysis of alpha2-, beta1- and AT2-abs in Q1 on log(Survival Time) via Large Artery Artherosclerosis Strokes**

Supplementary Figure 9: Causal mediation analysis of alpha2 adrenergic, beta 1 adrenergic and angiotensin II receptor type 2 (AT2) antibodies on log(survival time) via potential mediation through large artery atherosclerosis strokes. ACME = average causal mediation effect, ADE = average direct effect. Dashed line represents the corrected estimate while black line represents the raw estimate.
